# A transcriptome and proteome of the tick *Rhipicephalus microplus* shaped by the genetic composition of its hosts and developmental stage

**DOI:** 10.1038/s41598-020-69793-3

**Published:** 2020-07-30

**Authors:** Gustavo R. Garcia, José Marcos Chaves Ribeiro, Sandra Regina Maruyama, Luiz Gustavo Gardinassi, Kristina Nelson, Beatriz R. Ferreira, Thales Galdino Andrade, Isabel K. Ferreira de Miranda Santos

**Affiliations:** 1 Department of Biochemistry and Immunology, Ribeirão Preto School of Medicine, University of São Paulo, Avenida Bandeirantes 3900, Ribeirão Preto, SP 14049-900 Brazil; 2 Laboratory of Malaria and Vector Research, National Institute of Allergy and Infectious Diseases, National Institutes of Health, Bethesda, MD USA; 3 Center for the Study of Biological Complexity, Virginia Commonwealth University, Richmond, VA USA; 4 Department of Maternal-Child Nursing and Public Health, Ribeirão Preto School of Nursing, USP, Ribeirão Preto, SP Brazil; 5 Present Address: Superintendence of the São Paulo State Technical and Scientific Police, Ribeirão Preto, SP Brazil; 6 Present Address: Department of Genetics and Evolution, Center for Biological Sciences and Health, Federal University of São Carlos, São Carlos, SP Brazil; 7 Present Address: Institute of Tropical Pathology and Public Health, Federal University of Goiás, Goiânia, GO Brazil

**Keywords:** Gene expression profiling, Proteomics, Parasite immune evasion, Parasite biology, Parasite genomics, Vaccines

## Abstract

The cattle tick, *Rhipicephalus microplus*, is a monoxenous tick that co-evolved with indicine cattle on the Indian subcontinent. It causes massive damage to livestock worldwide. Cattle breeds present heritable, contrasting phenotypes of tick loads, taurine breeds carrying higher loads of the parasite than indicine breeds. Thus, a useful model is available to analyze mechanisms that determine outcomes of parasitism. We sought to gain insights on these mechanisms and used RNA sequencing and Multidimensional Protein Identification Technology (MudPIT) to generate a transcriptome from whole larvae and salivary glands from nymphs, males and females feeding on genetically susceptible and resistant bovine hosts and their corresponding proteomes. 931,698 reads were annotated into 11,676 coding sequences (CDS), which were manually curated into 116 different protein families. Male ticks presented the most diverse armamentarium of mediators of parasitism. In addition, levels of expression of many genes encoding mediators of parasitism were significantly associated with the level and stage of host immunity and/or were temporally restricted to developmental stages of the tick. These insights should assist in developing novel, sustainable technologies for tick control.

## Introduction

*Rhipicephalus microplus*, the cattle tick, is distributed worldwide^1,2^. Hematophagy and physical damage caused by the tick’s bites during a blood meal, as well as transmission of pathogens, including *Babesia ssp*. and *Anaplasma marginale* cause huge economic losses globally^3,4^. *R. microplus* is a monoxenous tick, consequently it spends its 3-week parasitic cycle on the same host, thus confronting all stages of its host’s immune responses^5^. Ticks use an array of molecules from their saliva to attach to their hosts and neutralize host inflammatory and immune responses and saliva also facilitates transmission of pathogens^6–8^. The current methods for controlling these ectoparasites use acaricides, which pollute the environment, contaminate food products and are selecting acaricide-resistant ticks^3^. Thus, development of new technologies for controlling *R. microplus* is urgent.

To date, two commercial vaccines, GAVAC and TickGARD, have been marketed to control cattle tick infestations^9,10^. Their design is based on the Bm86 antigen, a gut protease and a so-called “concealed” antigen^11,12^, i.e., an antigen to which hosts are not exposed to during natural infestations, as opposed to salivary antigens. While ticks feeding on cattle vaccinated with either of these vaccines do become damaged and present with significant reduced reproductive efficiency^9,10^, these vaccines require more boosts^13,14^ than are logistically and economically feasible for cattle producers^14,15^.

According to the proponents of the advantages of concealed over exposed antigens, the former would not be subjected to the tick’s immune evasion mechanisms^11,12^. This premise thus implies that tick salivary, “exposed” proteins are weakly or never immunogenic for a host and/or that any attempt by the host’s immune system to neutralize the proteins will be thwarted by their immunosuppressive actions. However, evidence shows that salivary antigens can be immunogenic for hosts: indeed, bovines presenting genetic resistance to ticks present higher titers of serum antibodies that react with more salivary proteins than bovines presenting genetic susceptibility to tick infestations^16,17^. Furthermore, long before the concept of concealed antigens brought into question the usefulness of exposed antigens, a pioneering study by William Trager showed that, in comparison to other extracts of tick tissues, those made with salivary glands were the most potent for promoting protective immunity against ticks^18,19^. The advent of DDT, soon after these studies were undertaken, possibly made immunobiological control of tick vectors of secondary interest. When resistance to acaricides finally arose, studies showing that immunity against salivary antigens decreased tick loads were again undertaken^20,21^. Another factor that favors salivary antigens in vaccines is the fact that persistent exposure to antigens is thought to be necessary to maintain immune memory^22^; thus, another advantage of employing salivary antigens instead of concealed antigens would be the natural boosts that cattle will receive when exposed to tick-infested pastures.

We recently demonstrated that a multicomponent vaccine formulated with salivary proteins from *R. microplus* significantly reduced tick loads in immunized cattle of a tick-susceptible breed and that this protection was accompanied by increased levels of antigen-specific IgG1 and IgG2 antibodies against two of the antigens; levels of antibodies were boosted during a challenge infestation^23^. Functional analyses of the gene expression profile of these hosts’ whole-blood leukocytes showed that vaccination induced responses associated with chemotaxis, cell adhesion, T-cell responses and wound repair^24^. However, the efficacy of this vaccine needs to be improved; among the many secreted salivary proteins that ticks produce, two were targeted, therefore, possibly more proteins are needed.

A thorough understanding of tick biology is needed to identify crucial targets, the limiting step in the production of any vaccine. As of this writing, over the last 15 years, more than forty studies (keywords for searches in PubMed: “transcriptome” plus the name of each genus of hard and soft ticks) have employed high throughput, “omic” analyses of tick tissues. They focused on tissues and morphs of various species of hard and soft ticks undergoing different stressors. Regarding *R. microplus*, these studies number fourteen and have examined molecular repertoires in different tissues (gut, synganglion, ovary, Gené’s organ and salivary glands or whole ticks), different morphs (larvae, nymphs, and/or adults) and conditions (unfed or feeding at different periods, infected or uninfected with pathogens, resistant or sensitive to acaricides and exposed to odors of/fed on tick-resistant and/or tick-susceptible breeds of cattle). Of these studies, three focused on the salivary glands of *R. microplus*: we examined a transcriptome generated with Sanger sequencing of transcripts from larvae and salivary glands of nymphs, male and female ticks feeding on genetically tick-resistant and susceptible breeds of cattle^23^; with a microarray built from expressed sequence tags (ESTs) from a transcriptome generated with Sanger sequencing of a pool of many tick tissues^26^, Mercado-Curiel and colleagues examined salivary glands from male ticks fed on uninfected or *Anaplasma marginale*-infected cattle of a tick-susceptible breed^25^; with MudPIT, Tirloni and colleagues generated the first proteome of cattle tick saliva and salivary glands from partially and fully fed females fed on a tick-susceptible breed of cattle^27^. However, none of these studies present sufficient information about which proteins to target with vaccines because not all possible targets were represented due to the molecular techniques and/or experimental design employed.

Part of the specificity of tick-host interfaces and of the reproductive success of the tick is explained by the complementarity between components of saliva from a given species of tick and the components of its preferred host’s inflammatory, hemostatic and immune defenses. In this context, success of the life cycle of *R. microplus* depends on the breed of the bovine host on which it feeds: loads of ticks in cattle vary according to breed^28^ and this variation is highly heritable^29^, therefore bovines offer a useful model to analyze the biological mechanisms that result in these phenotypes. Indicine cattle carry low ticks loads; they are thought to have reached a mutual agreement of boundaries with *R. microplus* because they co-evolved on the Indian subcontinent^30,31^. *R. microplus* reproduces after feeding on indicine cattle, albeit with lower fertility than when feeding on taurines^29^.

The role of the salivary constituents of *R. microplus* in resistance or susceptibility associated with different cattle breeds is not completely understood. The model of this study employs the indicine Nelore breed (*Bos taurus indicus*) as a resistant host and the taurine Holstein–Friesian breed (*Bos taurus taurus*) as susceptible host carrying hundreds of ticks. Their distinct local reactions to tick bites have been previously described at a histological and molecular level^32,33^. Herein we generate and describe a transcriptome and a proteome produced with deep sequencing and multidimensional protein identification technology (MudPIT), respectively, of larvae and of salivary glands from nymphs and adult *R. microplus* feeding on Nelore and Holstein–Friesian breeds of bovines.

## Results and discussion

### The annotated transcriptome and proteome of *R. microplus*

#### Overall characteristics

The salivary glands of ticks are the main repository of their mediators of parasitism. In the present study we sought to gain insights on the parasitic mechanisms deployed by saliva from a monoxenous (i.e., single host) tick during its development from unfed larvae, to nymphs, to adults (males and females), while sequentially confronting innate and then acquired host defenses of genetically tick-resistant and tick-susceptible bovines. We also sought to understand if the differences in tick loads seen between taurine and indicine cattle are reflected in the sialotranscriptome, and if and how they correspond to the distinct composition of these hosts’ responses to ticks, previously described by us and others^32,33^. By employing high-throughput approaches such as large scale 454 Sequencing parallel pyrosequencing for RNA and MudPIT, we recovered approximately 1 million sequences, 11,676 CDS and 321 different proteins, respectively. Many of these transcripts and proteins are associated with tick parasitic behavior, thus provide better understanding about the developmental biology of *R. microplus* in the context of its hosts’ defenses. The study corroborates the high resolution proteome generated by Tirloni and colleagues^27^ and complements it with information from nymphs and males.

In order to follow the parasitic cycle of *R. microplus*, feeding nymphs, males and females were collected from an artificial infestation of Nelore and Holstein bovines; these morphs supplied salivary glands (SGs) and, in the case of engorged females, the eggs masses from which the larvae studied herein ecloded. For 454 sequencing and corresponding MudPIT proteomes we generated eight libraries named according to the host (tick-susceptible, ‘S’ or tick-resistant, ‘R’) on which ticks were feeding and to the morphs the SGs were dissected from, as follows: two libraries and corresponding proteomes from unfed larvae obtained from *R. microplus* engorged females fed on tick-resistant or tick-susceptible bovine hosts (UFLR and UFLS, respectively); two libraries and corresponding proteomes from SGs dissected from nymphs (NSGR and NSGS, respectively); two libraries and corresponding proteomes from female SGs (FSGR and FSGS, respectively); and two libraries and corresponding proteomes from male SGs (MSGR and MSGS, respectively). In addition, two proteomes were generated with saliva collected from semi-engorged females removed from tick-resistant or tick-susceptible hosts (SalivaR and SalivaS, respectively).

#### The transcriptome

The pyrosequencing of eight salivary *R. microplus* libraries resulted in a total of 2,413,950 reads, which were assembled into 348,707 contigs, including singletons. Next, the assembled data was filtered by clustering contigs with at least 4 mapped reads and larger than 150 nucleotides (nt), a step which retrieved 1,999,086 reads assembled into 40,734 contigs (approximately 83% of the total reads generated). Subsequent clustering resulted in the extraction of 11,676 putative CDS, which were derived from 931,698 reads (Table 1 and Supplemental File 1). The libraries of *R. microplus* fed on tick-resistant hosts resulted in 53% of the total of reads, while the remaining reads were recovered from libraries of ticks fed on tick-susceptible hosts (Table 1). The MSGR library presented the greatest number of reads (254,657), followed by NSGR, FSGR and UFLR. In libraries derived from ticks fed on tick-susceptible hosts, the NSGS presented the largest number of reads, followed by MSGS, FSGS and UFLS (Table 1).

**Table 1:** Abundance and distribution of reads and coding sequences from the sialotranscriptome of *Rhipicephalus microplus*.

Abundance and distribution of transcripts by library				
Library	RmR*		RmS	
	N° of CDS	N° of Reads	N° of CDS	N° of Reads
UFL	6,018	54,115 (5.8%)	3,755	86,135 (9.2%)
NSG	7,060	119,574 (12.8%)	5,425	151,406 (16.2%)
MSG	9,412	254,657 (27.3%)	9,215	106,691 (11.4%)
FSG	7,305	70,136 (7.5%)	2,238	88,984 (9.5%)
Total	11,281	498,482	10,667	433,216

Abundance and distribution of transcripts by class				
Class	N° of CDS	N° of Reads	% of total reads	Reads/CDS
Secreted	3,600	278,351	29.88	77.3
Housekeeping	4,629	307,184	33.97	66.4
Unknown	2,822	331,738	35.61	117.6
Transposable elements	618	14,139	1.52	22.9
Viral	7	286	0.03	40.9
Total	11,676	931,698		

*RmR libraries constructed with *R. microplus* ticks fed on resistant hosts, *RmS* libraries constructed with ticks fed on susceptible hosts, *UFL* unfed larvae derived from engorged female ticks, *NSG, MSG, FSG* salivary glands from feeding nymphs, males and females, respectively.

**Figure 1:**
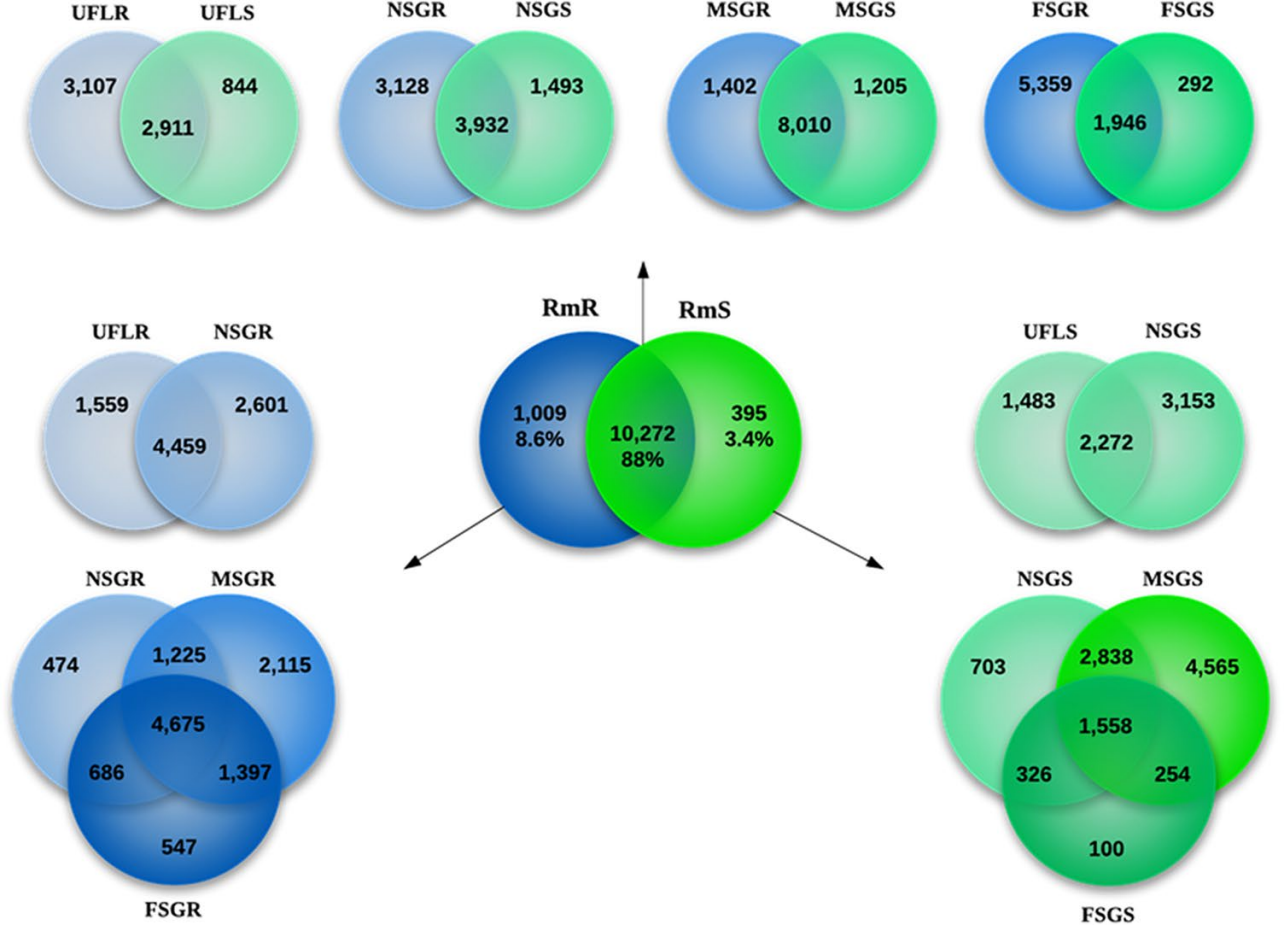
The sialotranscriptome of *Rhipicephalus microplus* according to its developmental stage and source of blood meal. The Venn diagrams show the number of common and unique CDS in unfed larvae and salivary glands of nymphs, females and males of R. microplus feeding on resistant or susceptible bovine hosts (green and blue circles, respectively). Abbreviations for libraries that generated the transcriptome: *UFLR and UFLS* unfed larvae ecloded from eggs oviposited by engorged female ticks fed on resistant or susceptible hosts, respectively, *NSGR and NSGS* salivary glands obtained from nymphs ticks fed on resistant or susceptible hosts, respectively, *MSGR and MSGS* salivary glands obtained from male ticks fed on resistant or susceptible hosts, respectively, *FSGR and FSGS* female salivary glands obtained from partially fed female ticks fed on resistant or susceptible hosts, respectively. The diagram at the center of the figure, "RmR vs RmS", represents the 11,676 CDS generated by all eight libraries from *R. microplus*. The diagrams at the top of the figure represent distributions of CDS according to the developmental stages of the tick and the origin of its blood meal. The diagrams at the left and right of the figure represent distributions of CDS according to developmental stages of ticks when fed on resistant and susceptible hosts, respectively.

Automatic annotation, as previously reported^23^, is described in the Methods section. The 11,676 annotated CDS were classified into five main categories named putatively secreted (S), housekeeping (H), unknown (U), transposable elements (TE) and viral (V) (Table 1 and Supplemental Files 1 and 2). Approximately 30% of the reads were annotated into 3,600 CDSs presenting with a predicted signal peptide and/or significant matches with previously identified secreted proteins and were classified in the S category; 34% of reads were annotated into the H category due to a high similarity with constitutive genes of arthropods, including ticks. Most of the reads (about 36%) were annotated as unknown proteins because they did not present significant matches with any known protein sequence (Table 1). The categorized CDS were then organized into 116 families according to their putative functional classes, which allowed for independent comparisons between developmental stages of *R. microplus* and/or between the effect of host backgrounds upon the salivary functions and protein repertoires (Supplemental Files 1 and 2). Of interest, the comparative analysis between all libraries from *R. microplus* showed that 88% of CDSs were common to and expressed by all forms of the ticks, regardless of source blood meal or developmental stages. On the other hand, the expression of 12% of the CDS was affected by the breed (i.e., genetic background) of the tick’s bovine hosts (Fig. 1): 8.6% of the CDS were found exclusively in *R. microplus* ticks fed on tick-resistant bovine hosts, being more than twice the amount of CDS, 3.4%, found exclusively in ticks fed on tick-susceptible hosts (Fig. 1, central and upper Venn diagrams). This finding indicates that the differences in tick loads seen between cattle breeds may be caused by complementary repertoires of salivary mediators of parasitism and host responses. As expected, we found significant differences in transcriptional profiles between instars of *R. microplus* during the whole infestation period on hosts of the same breed (Fig. 1—left and right columns of Venn diagrams and Supplemental File 1).

#### The proteome

**Figure 2:**
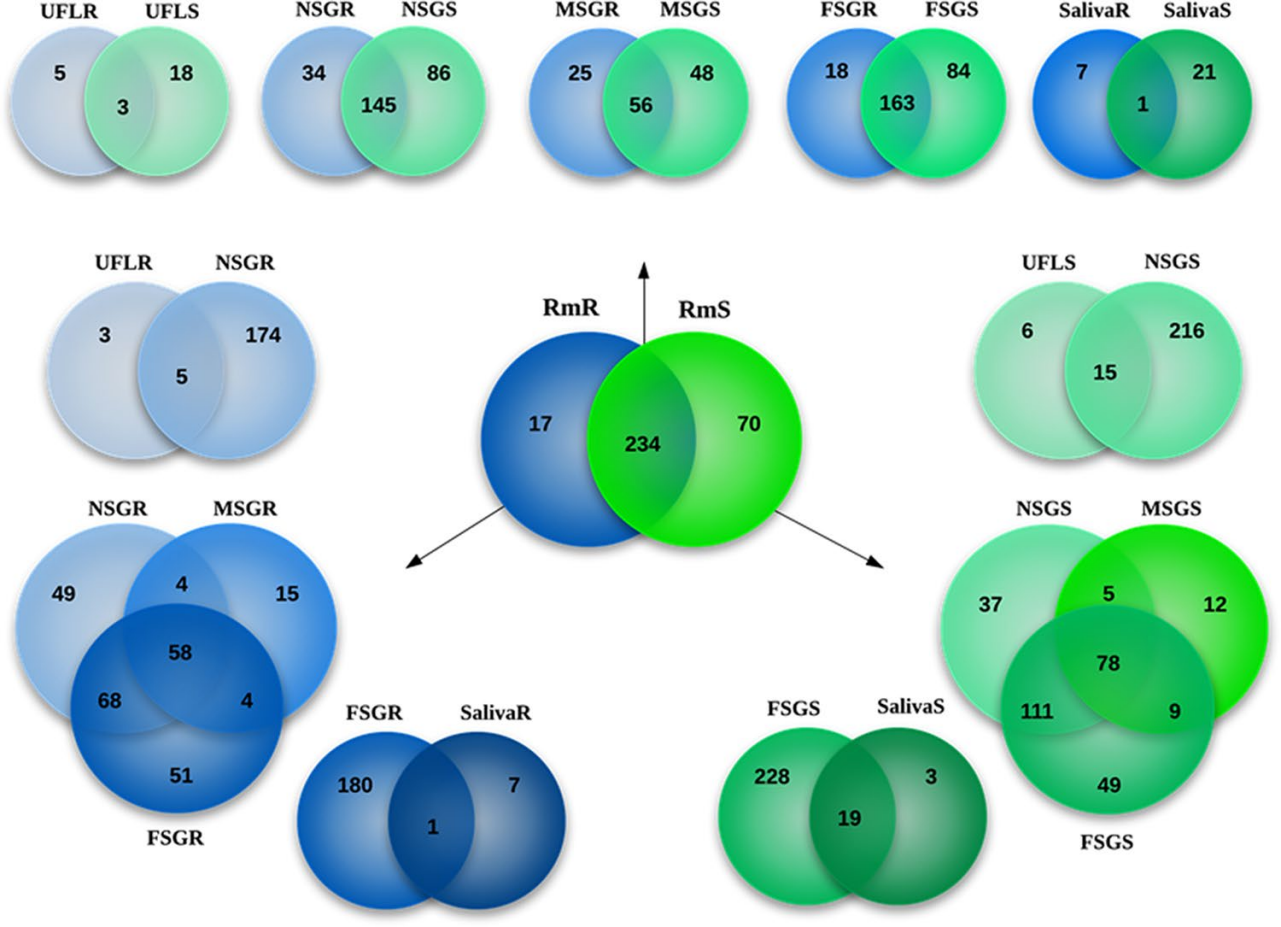
The proteome of larvae, salivary glands and saliva of *Rhipicephalus microplus* according to its developmental stage and source of blood. The Venn diagrams show the number of common and unique proteins identified in unfed larvae, salivary glands and saliva of R. microplus feeding on resistant or susceptible bovine hosts (green and blue circles, respectively). Abbreviations: *UFLR and UFLS* unfed larvae ecloded from eggs oviposited by engorged female ticks fed on resistant or susceptible hosts, respectively, *NSGR and NSGS* salivary glands obtained from nymphs ticks fed on resistant or susceptible hosts, respectively, *MSGR and MSGS* salivary glands obtained from male ticks fed on resistant or susceptible hosts, respectively, *FSGR and FSGS* female salivary glands obtained from partially fed female ticks fed on resistant or susceptible hosts, respectively, *SalivaR and salivaS* saliva obtained from semi-engorged female ticks fed on resistant or susceptible hosts, respectively. The diagram at the center of the figure, "RmR vs RmS", represents the 32 l proteins different proteins identified in all materials. The diagrams at the top of the figure represent distributions of proteins according to the developmental stages of the tick and the origin of its blood meal. The diagrams at the left and right of the figure represent the distributions of proteins according to developmental stages of the tick when fed on resistant and susceptible hosts, respectively.

Next, we generated a proteome from ten different materials (salivary glands, saliva and extracts of larvae) from *R. microplus* in distinct developmental stages and fed on hosts with distinct genetic backgrounds, in which resultant protein sequences were identified with BLAST using as a database our annotated catalogue obtained with the transcriptome (BioProject ID PRJNA329522). We identifed a total of 321 different proteins (Fig. 2 and Supplemental File 3): 304 different proteins were present in materials derived from ticks fed on susceptible hosts (70 of them unique to ticks feeding on susceptible hosts), whereas 251 proteins were present in materials derived from ticks fed on resistant hosts (17 of them were unique to ticks feeding on resistant hosts) (Fig. 2 and Supplemental File 3); 234 proteins were identified in all of the materials from *R. microplus* (Fig. 2, central Venn diagram). We retrieved sequences of proteins that were classified into 42 families out of the 116 protein families annotated in the corresponding sialotranscriptome (Supplemental File 2, column titled "Match with Proteomes"). Most of the MudPIT-identified proteins matched with CDSs from H protein families. However, we detected proteins that possibly mediate parasitism of *R. microplus* in families of the S category (Supplemental File 3). Among these, we highlight serpins, serine proteases, lipocalins, antigen 5 proteins, neutrophil elastase inhibitors, immunoglobulin-binding proteins (IGBP), glycine-rich proteins and other secreted proteins (Supplemental File 3).

#### Relative abundance of transcripts in protein families

**Figure 3:**
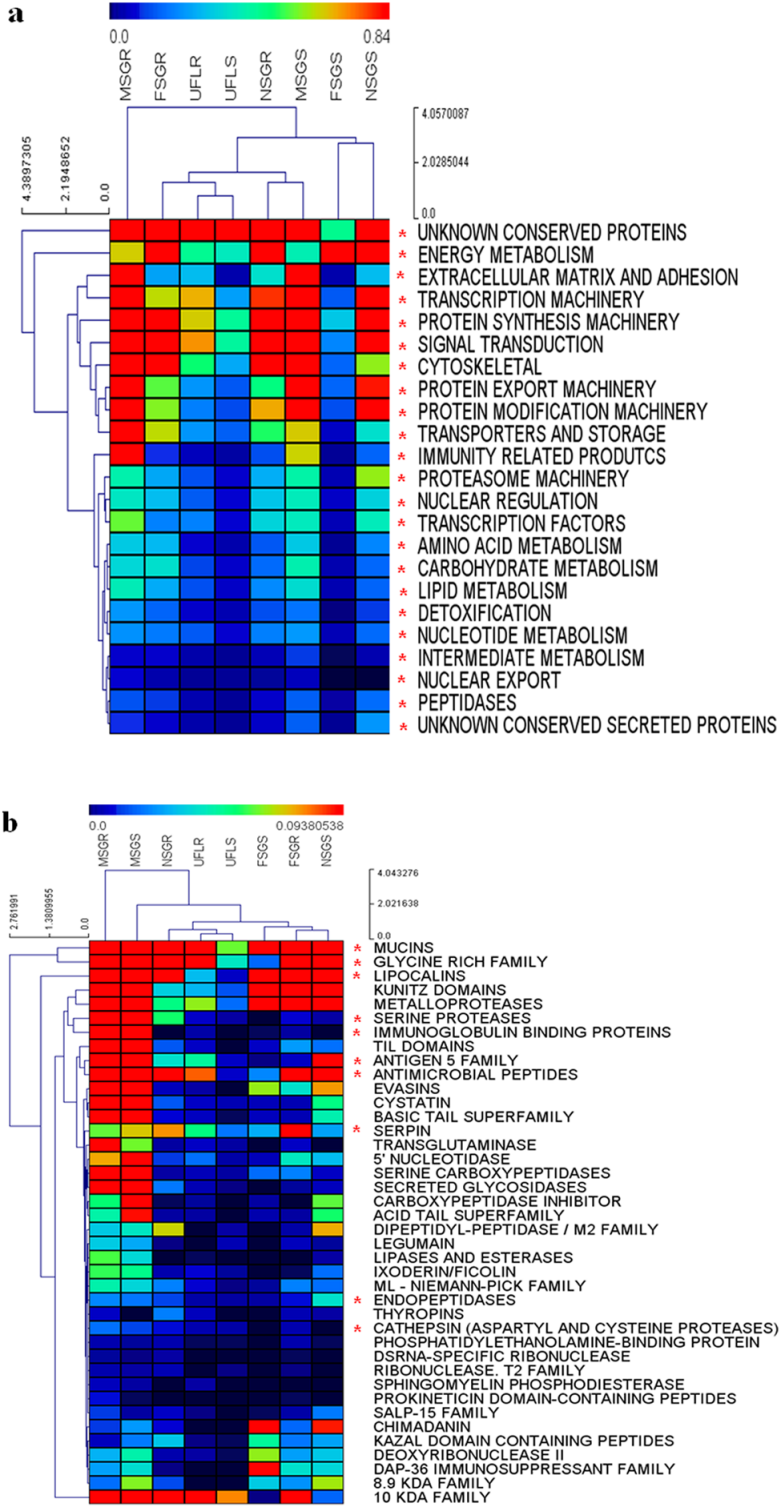
Hierarchical clustering of putative protein families from the transcriptome of *Rhipicephalus microplus*. Heatmaps display the levels of expression of the protein families (rows) for each library (columns). (**a**) Families classified in housekeeping category. (**b**) Families classified in secreted category. The color scale represents the log_2_ transformation of % of reads + 1. The transition of colors from blue to red represents an increase in the % of reads. Black means % of reads < 0.00001 and red means % of reads ≥ 3.68. The length of branches in hierarchical trees indicates the degree of similarity between objects, either libraries (columns) or protein families (rows). Side scales represent the node height (cluster-to-cluster distance). *UFLR and UFLS* unfed larvae ecloded from eggs oviposited by engorged female ticks fed on resistant or susceptible hosts, respectively, *NSGR and NSGS* salivary glands obtained from nymphs fed on resistant or susceptible hosts, respectively, *MSGR and MSGS* salivary glands obtained from male ticks fed on resistant or susceptible hosts, respectively, *FSGR and FSGS* female salivary glands obtained from partially fed female ticks fed on resistant or susceptible hosts, respectively. The clustering method used Euclidian distance with average linkage. Asterisks (*) indicate the family annotated in the transcriptome that matches with proteins identified in the proteome; all housekeeping families presented members in the proteome.

The majority of CDS annotated into the 116 protein families represented in the transcriptome (including the 42 protein families identified in the proteome) were shared between larval, nymph and adult instars of *R. microplus* (Figs. 1 and 2 and Supplemental File 2). To obtain further insights into the dynamics of transcription of CDS encoding those molecules, we performed a hierarchical clustering analysis employing the abundance of transcripts (relative percentage of reads; Fig. 3).

The 23 housekeeping protein families, including unknown conserved protein families, were clustered in groups according to their levels of expression (Fig. 3a). The unknown conserved protein family was the most abundant among all libraries, followed by energy metabolism, transcription machinery, protein synthesis, signal transduction, cytoskeleton, protein export and modification machinery families. The remaining H protein families presented low expression. Except for the libraries of UFLR and UFLS and of NSGS and FSGS, which clustered together, we were unable to observe a clustering pattern according to the tick’s developmental stages or source of its blood meal. Of note was the MSGR library, which did not cluster with any other library (Fig. 3b), first column at the left of the dendrogram), indicating that the patterns of expression of this library were very different from those of the other libraries. In addition, the MSGR library presented 10 highly expressed protein families, including products related to immunity. The MSGR library also contained the greatest number of transcripts (27.3% of total reads) when compared to all other libraries, including libraries derived from ticks fed on tick-susceptible hosts (Table 1). These transcriptional profiles demonstrate that the repertoire of salivary proteins changes according to life stage, as well as to the level of immunity in the source of host blood. In addition, all housekeeping protein families include members that were also identified in the proteome (Supplemental File 2, column "Match with proteomes?"; Fig. 3a, asterisks to left of name of protein family).

The S category presented a smaller amount of reads and, consequently, CDS when compared to the H category (Table 1 and Supplemental Files 1 and 2). Nevertheless, this category possesses diverse protein families, such as enzymes, nucleases, antimicrobial peptides (AMPs) and others (Supplemental Files 1 and 2) that are predicted to assist the tick in all its parasitic life stages. Hierarchical clustering using levels of expression from putative S protein families also showed a distinct pattern of distribution among protein families or *R. microplus* libraries (Fig. 3b). The mucin and glycine-rich families were clustered together and presented high levels of expression in almost all libraries. There were two notable exceptions: libraries UFLS and FSGS, which presented less transcripts for glycine-rich proteins. That these particular stages apparently depend less on these proteins than other stages need further investigations in order to fully explain the biology of this species of tick. In the same manner, lipocalins, kunitz domain-containing proteins, metalloproteases, proteins of the 10 KDa family and serine proteases were also highly expressed in almost all libraries. Of note, several of the protein families classified into the S category were identified in the proteome (asterisks at the left of names of the protein family in Fig. 3b; Supplemental Files 2 and 3). As observed for heatmaps displaying the levels of expression in H protein families, clusters based on developmental stages were formed only by libraries from unfed larvae, regardless of their host’s background (Fig. 3b, upper dendogram). Of interest, transcripts coding for proteins in the S family from nymphs fed on tick-susceptible hosts were clustered together with transcripts from female ticks fed on tick-resistant hosts (Fig. 3b, upper dendogram). This may signify that the immune response of resistant hosts delays development of females feeding on them. Independently of the source of the blood meal, libraries from male ticks are those that present the highest levels of expression compared to all other instars (Table 1, Fig. 3 and Supplemental File 1).

### The genetic composition of the host affects transcriptional profiles in *R. microplus*

The transcriptional and protein profiles of the SGs in all developmental stages of the tick were strongly affected by the source of the blood meal, whether a genetically tick-resistant or tick-susceptible host (i.e., in this study the Nelore or Holstein breed, respectively; Figs. 1, 2, 3; Supplemental Files 1, 2, 3; results of chi-square analyses in columns AD-AH of Supplemental File 1). Furthermore, practically all protein families have CDSs that were differentially expressed between libraries derived from either tick fed on tick-resistant Nelore or tick-susceptible Holstein hosts.

**Figure 4:**
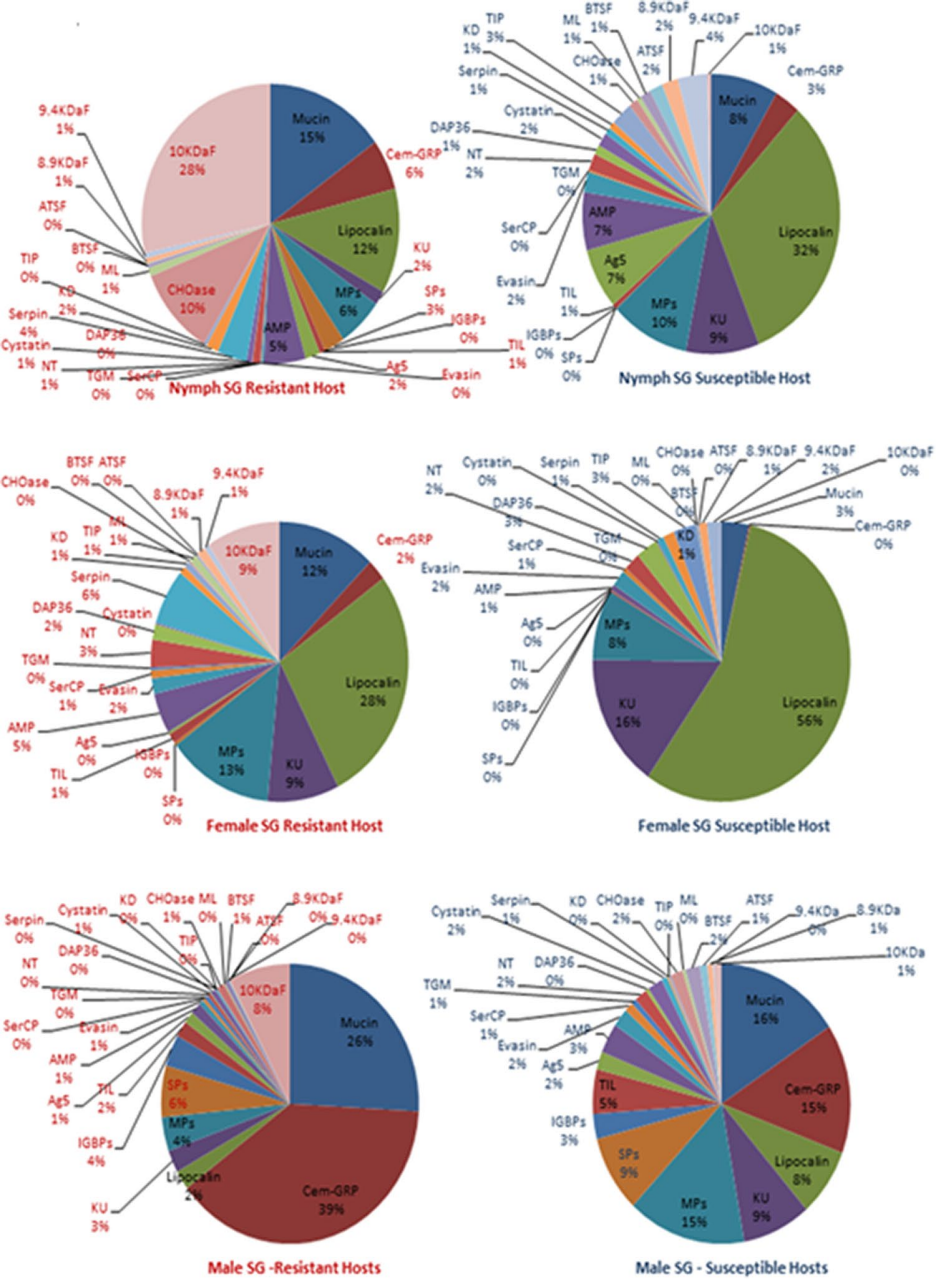
Representation of the most transcribed CDS encoding secreted proteins with putative functions in parasitism. Representation of protein families was expressed as a percentage of the transcripts from the total found for all dominant families in NSGS, NSGR, FSGS, FSGR, MSGS and MSGR libraries. Abbreviations and description of protein families: *Cem-GRP* cement-like, glycine-rich proteins, *KU* Kunitz domain-containing protease inhibitors, *MP* metalloproteases, *SP* serine proteases, *IGBPs* immunoglobulin-binding proteins, *TIL* trypsin inhibitor-like proteins, *Ag5* antigen 5 anti-oxidant proteins, *AMP* antimicrobial peptides, *Evasin* chemokine-binding proteins, *SerCP* serine carboxypeptidases, *TGM* transglutaminase, *NT* nucleotidadases, *DAP36* DAP 36 immunosuppressor, *KD* Kazal domain-containing proteins, *TIP* Chimadanin thrombin inhibitors, *CHOase* digestive carbohydrases, *ML* ML-domain-containing proteins, *BTSF* basic tail protein superfamily, *ATSF* acid tail protein superfamily, *8.9KDa* 8.9 KDa protein family, *9.4KDa* 9.4 KDa protein family, *10KDa* 10 KDa protein family.

Among the protein families annotated as secreted and presenting putative functions in parasitism, twenty-six were represented in salivary glands from nymphs, females and males by between at least 300 up to almost 28,000 transcripts (Supplemental File 1). In order to understand how these dominant protein families are being recruited when the cattle tick is confronted by different types of host immunity, we examined the representation of each, expressed as percentages of transcripts from the total found for these dominant families in each library (pie charts in Fig. 4) and if their distribution was random or biased towards the source of blood, i.e., whether from resistant indicines (Nelore breed in this study) or susceptible taurines (Holstein–Friesian breed in this study) cattle (Table 2). The data indicate that in order to feed on resistant hosts both nymphs and adults (males and females) seem to rely significantly more on mucins, cement-like proteins, serpins, digestive carbohydrases (at the nymphal stage) and on the 10 KDa family of proteins compared to ticks feeding on susceptible hosts. Conversely, ticks feeding on resistant hosts seem to rely significantly less on lipocalins, proteinase inhibitors containing Kunitz domains, metalloproteases, Antigen 5 anti-oxidants, anti-microbial peptides, chemokine-binding evasins, serine carboxypeptidades, nucleotidases, immunosuppressors of the DAP36 family, cystatins, Kazal domain-containing proteins, chimadanin-type thrombin inhibitors and proteins of the basic tail, acid tail, 8.9 KDa and 9.4 KDa families. However, the gene expression profiles observed in ticks feeding on resistant hosts may not represent an adaptation to their particular immune responses, but rather a “sialopathology”, i.e., a response of the tick’s salivary glands that is detrimental to its life cycle. In other words, producing too much or too little of one or more of salivary proteins when feeding on resistant hosts damages the tick, is a maladapted response and explains its lower reproductive efficiency in this situation.

**Table 2:** Representation of transcripts coding for families of secreted proteins in libraries of salivary glands from nymphs, females and males of *R. microplus* ticks according to source of blood, whether genetically resistant or susceptible bovine hosts.

Library	N° of reads		Library	N° of reads		χ^2^ test *P value*	Library	N° of reads		Library	N° of reads		χ^2^ test *P value*
	Observed	Expected		Observed	Expected			Observed	Expected		Observed	Expected	
**Mucins**							**Glycine-rich cement-like proteins**						
NSGR	**632	517	NSGS	522	636	45.89 < *0.001*	NSGR	**246**	205	NSGS	213	253	14.06 < *0.001*
FSGR	**543**	341	FSGS	230	431	212.52 < *0.001*	FSGR	**108**	53	FSGS	12	67	100.72 < *0.001*
MSGR	**15,946**	13,463	MSGS	2,974	5,456	1669.40 < *0.001*	MSGR	**23,955**	19,232	MSGS	2,839	7,561	4,412.028 < *0.001*
**Lipocalins**							**Kunitz-domain containing proteins**						
NSGR	508	1,158	NSGS	**2,103**	1,452	666.57 < *0.001*	NSGR	69	294	NSGS	**600**	374	308.22 < *0.001*
FSGR	1,213	2,163	FSGS	**3,764**	2,813	761.75 < *0.001*	FSGR	394	633	FSGS	**1,046**	807	161.22 < *0.001*
MSGR	1,371	2012	MSGS	**1,491**	850	691.69 < *0.001*	MSGR	1582	2,257	MSGS	**1629**	954	684.31 < *0.001*
**Metalloproteases**							**Serine Proteases**						
NSGR	240	393	NSGS	**652**	498	106.27 < *0.001*	NSGR	**109**	49	NSGS	3	62	126.33 < *0.001*
FSGR	**585**	499	FSGS	545	631	26.62 < *0.001*	FSGR	**15**	7	FSGS	0	8	16.83 < *0.001*
MSGR	2,425	3,676	MSGS	**2,817**	1566	1,444.92 < *0.001*	MSGR	3,671	3,769	MSGS	1679	1581	8.65 *0.003†*
**Immunoglobulin G-binding proteins**							**Trypsin-like inhibitor domain-containing proteins**						
NSGR	0	0	NSGS	0	0	N.S. *	NSGR	29	29	NSGS	36	37	N.S
FSGR	0	0	FSGS	0	0	N.S	FSGR	**50**	28	FSGS	13	35	30.40 < *0.001*
MSGR	**2,251**	1974	MSGS	548	824	131.80 < *0.001*	MSGR	1,145	1,491	MSGS	**973**	627	271.330 < *0.001*
**Antigen 5**							**Anti-microbial peptides**						
NSGR	78	236	NSGS	**458**	300	188.63 < *0.001*	NSGR	220	290	NSGS	**438**	367	30.01 < *0.001*
FSGR	13	7	FSGS	2	8	9.38 *0.002†*	FSGR	**205**	110	FSGS	44	139	145.99 < *0.001*
MSGR	829	860	MSGS	392	360	N.S	MSGR	848	1,058	MSGS	**655**	445	141.234 < *0.001*
**Chemokine-binding evasins**							**Serine carboxypeptidases**						
NSGR	11	73	NSGS	**154**	92	92.35 < *0.001*	NSGR	3	7	NSGS	13	9	N.S
FSGR	78	91	FSGS	128	115	N.S	FSGR	42	34	FSGS	35	43	N.S
MSGR	337	520	MSGS	**402**	219	217.84 < *0.001*	MSGR	221	304	MSGS	210	127	75.30 < *0.001*
**Transglutaminases**							**Nucleotidases**						
NSGR	4	2	NSGS	4	2	N.S	NSGR	41	74	NSGS	**128**	94	26.24 < *0.001*
FSGR	13	6	FSGS	0	7	14.30 < *0.001*	FSGR	141	126	FSGS	138	153	4.46 *0.035†*
MSGR	297	297	MSGS	124	124	N.S	MSGR	235	377	MSGS	**301**	158	181.0 < *0.001*
**DAP36 immunosuppressor**							**Cystatins**						
NSGR	2	33	NSGS	**73**	42	50.61 < *0.001*	NSGR	29	57	NSGS	**101**	73	24.21 < *0.001*
FSGR	78	117	FSGS	**187**	148	22.43 < *0.001*	FSGR	8	8	FSGS	10	10	N.S
MSGR	52	90	MSGS	**76**	37	53.35 < *0.001*	MSGR	414	544	MSGS	**358**	228	104.20 < *0.001*
**Serpins**							**Kazal Domain-containing proteins**						
NSGR	**156**	92	NSGS	53	117	77.63 < *0.001*	NSGR	65	52	NSGS	53	66	5.31 *0.021†*
FSGR	**283**	240	FSGS	57	100	25.98 < *0.001*	FSGR	38	94	FSGS	96	40	112.134 < *0.001*
MSGR	121	186	MSGS	**143**	78	75.771 < *0.001*	MSGR	11	36	MSGS	40	15	56.28 < *0.001*
**Chimadanin thrombin inhibitors**							**Digestive Carbohydrases**						
NSGR	16	86	NSGS	**179**	109	100.54 < *0.001*	NSGR	**430**	223	NSGS	74	281	344.40 < *0.001*
FSGR	34	100	FSGS	**192**	126	76.02 < *0.001*	FSGR	7	4	FSGS	1	5	4.48 0.034**†**
MSGR	21	49	MSGS	**49**	21	53.16 < *0.001*	MSGR	606	637	MSGS	298	267	4.96 *0.026†*
**ML Domain-containing proteins**							**Basic Tail superfamily proteins**						
NSGR	42	34	NSGS	36	44	N.S	NSGR	17	46	NSGS	**88**	59	32.10 < *0.001*
FSGR	**42**	21	FSGS	5	26	37.27 < *0.001*	FSGR	8	8	FSGS	11	11	N.S
MSGR	90	115	MSGS	**73**	48	17.50 < *0.001*	MSGR	323	467	MSGS	**341**	196	150.62 < *0.001*
**Acid Tail superfamily proteins**							**8.9 KDA family proteins**						
NSGR	4	50	NSGS	**109**	63	73.84 < *0.001*	NSGR	26	70	NSGS	**132**	88	47.93 < *0.001*
FSGR	4	5	FSGS	7	6	N.S	FSGR	47	51	FSGS	68	64	N.S
MSGR	91	192	MSGS	**181**	80	177.12 < *0.001*	MSGR	36	114	MSGS	**126**	48	178.80 < *0.001*
**9.4 KDA family proteins**							**10 KDA family proteins**						
NSGR	27	118	NSGS	**240**	149	123.81 < *0.001*	NSGR	**1,187**	541	NSGS	33	679	1,388.86 < *0.001*
FSGR	28	65	FSGS	**120**	83	36.96 < *0.001*	FSGR	**407**	181	FSGS	2	228	505.98 < *0.001*
MSGR	4	18	MSGS	**22**	8	35.31 < *0.001*	MSGR	**4,751**	3,497	MSGS	187	1,440	1,560.48 < *0.001*

^†^Not significant after Bonferroni correction; *N.S.: not significant; **: numbers in bold indicate that transcripts for the family are significantly overrepresented in the library.

On the other hand, ticks feeding on susceptible hosts contain a more abundant and diverse repertoire of inhibitors of host defenses. Moreover, the relative distribution of transcripts seen in females feeding on Nelores resembles that of SGs from nymphs feeding on Holsteins, suggesting, as already mentioned, that development of females is delayed when they feed on resistant hosts.

### Transcriptional and protein profiles in morphs of the cattle tick

The cattle tick’s larvae metamorphose to nymphs after approximately one week of feeding on their hosts and in another week metamorphose to adults, at which point males and females both feed on blood for approximately seven more days before the fertilized female releases and drops from the bovine host to the soil, generally in a pasture where its hosts graze^5^. During this time frame ticks must counteract different stages of their hosts’ innate and acquired defenses. The transcriptional and protein profiles (Fig. 3 and Supplemental Files 1, 2, 3) reveal that numerous CDS encoding protein families that participate in constitutive cellular functions and that assist parasitism are more highly or even exclusively expressed in salivary glands of male ticks. As can be seen in Fig. 3b, the MSGR and MSGS libraries contain at least 17 protein families in the Secreted category that are highly expressed and potentially directly associated with parasitism, including modulation of host defenses. We verified if distribution of reads for protein families in ticks feeding on susceptible hosts was random or biased towards a developmental stage. The results of these analyses are presented in Fig. 4 and Table 3 and suggest that for parasitism, nymphs and males rely on most of these families, being that, compared with males, nymphs recruit more lipocalins, chimadanin-type thrombin inhibitors and members of the 9.4 KDa protein family. Transcripts encoding lipocalins were the most abundant of the protein families in libraries from female SGs and were overrepresented in this stage, as were those encoding Kunitz domain-containing protease inhibitors, DAP36-type immunosuppressors, chimadanin-type thrombin inhibitors and the 9.4 KDa protein family. Interestingly, nymphs and females hardly rely on serine proteases, immunoglobulin G-binding proteins (IGBPs; transcripts encoding IGBPs were found almost exclusively in male ticks), serine carboxypeptidases and transglutaminases (also found almost exclusively in male ticks). Furthermore, compared with nymphs and males, females rely weakly on Antigen 5 anti-oxidant proteins, cement-type glycine-rich proteins, cystatins, proteins with trypsin inhibitor-like domains, digestive carbohydrases, ML-domain proteins and the basic tail, acid tail and 10 KDa families.

**Table 3:** Representation of transcripts coding for the main families of secreted proteins in salivary glands of nymphs, females and males of *R. microplus* ticks feeding on susceptible hosts.

Protein Family	N° of reads in Library						χ^2^ test; *P value*
	NSGS		FSGS		MSGS		
	Observed	Expected*	Observed	Expected	Observed	Expected	
Mucins	552	1627	230	955	**2,974**	1,174	4,063,636; < *0.001*
Cement-like glycine-rich proteins	213	1,327	12	779	**2,839**	958	5,427,113; < *0.001*
Lipocalins	2,103	3,187	**3,764**	1925	1,491	2,246	2,428,371; < *0.001*
Kunitz-domain containing proteins	600	1,421	**1,046**	842	**1629**	1,013	907,726; < *0.001*
Metalloproteases	652	1738	545	1,023	**2,817**	1,252	2,892,116; < *0.001*
Serine proteases	3	730	0	429	**1679**	523	3,729,824; < *0.001*
Immunoglobulin G-binding proteins	0	239	0	140	**548**	169	1,230,352; < *0.001*
Trypsin inhibitor-like proteins	37	445	13	262	**973**	316	1978,723; < *0.001*
Antigen 5 antioxidant enzymes	**458	372	2	218	**392**	262	298,833; < *0.001*
Anti-microbial peptides	438	496	44	291	**655**	351	482,189; < *0.001*
Chemokine-binding evasins	154	299	129	176	**402**	211	255,856; < *0.001*
Serine carboxypeptidases	13	112	35	66	**210**	79	317,645; < *0.001*
Transglutaminases	0	54	0	32	**124**	38	279,165; < *0.001*
Nucleotidases	128	247	138	145	**301**	175	149,759; < *0.001*
DAP36 immunosuppressors	73	147	**187**	86	76	103	161,951; < *0.001*
Cystatins	101	204	10	120	**358**	144	469,574; < *0.001*
Serpins	53	110	57	65	**143**	78	85,396; < *0.001*
Kazal domain-containg proteins	53	82	96	48	40	58	62,749; < *0.001*
Chimadanin thrombin inhibitor proteins	179	183	**192**	108	49	129	115,665; < *0.001*
Digestive Carbohydrases	74	163	1	96	**298**	115	434,333; < *0.001*
ML domain-containing proteins	36	50	5	29	**73**	35	64,956; < *0.001*
Basic Tail superfamily	88	192	11	113	**341**	136	460,102; < *0.001*
Acid Tail superfamily	109	130	7	76	**181**	91	154,033; < *0.001*
8.9 KDA family	132	142	68	84	**126**	100	10,263; < *0.006†*
9.4 KDA family	**240**	167	**120**	98	22	117	114,740; < *0.001*
10 KDa family	33	99	2	57	**187**	68	301,372; < *0.001*
Total reads in library	151.406		88.984		106.691		

*: Data are for a 2 X 2 contingency table; **: numbers in bold indicate that transcripts for the family are significantly overrepresented in the library; †Not significant after Bonferroni correction.

### Representation of specific protein families in the context of blood source and developmental stages of *R. microplus*

#### Glycine-rich proteins (GRPs) and mucins

**Figure 5:**
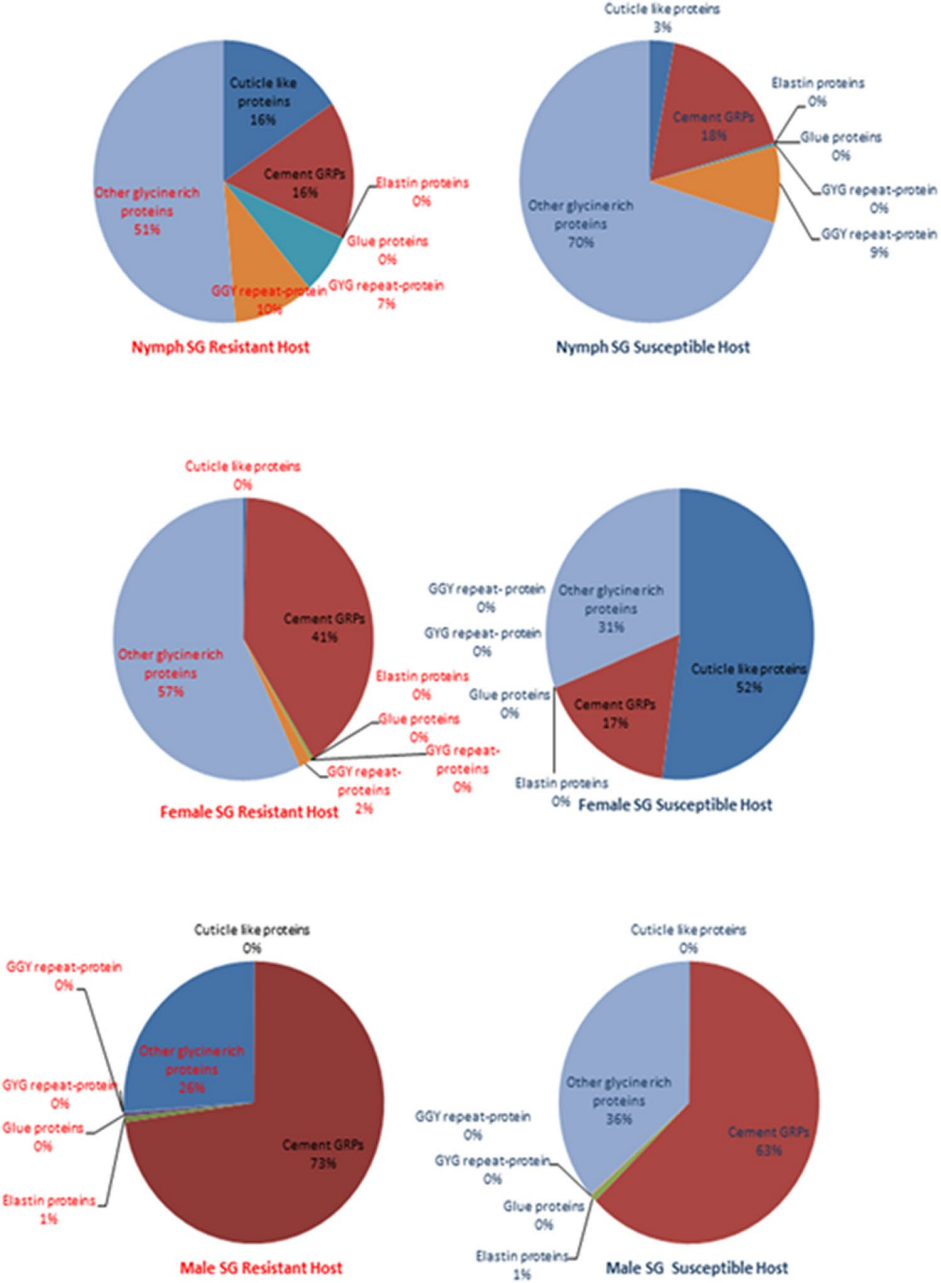
Representation of the most transcribed CDS encoding GRPs and mucins. The representation of each protein family was expressed as a percentage of the transcripts from the total found for all dominant families in each library from nymphs, females and males feeding on genetically tick-resistant and susceptible bovine hosts.

*Representation of GRPs according to developmental stage—*Because it is the most represented family, with a total of 41,435 transcripts as noted above, we also assessed the glycine-rich protein family, composed of five main subfamilies and that include the cement proteins. Previous work showed that this protein family is expressed in larger quantities and diversity of CDS in salivary glands of female *R. microplus* ticks as compared to those from females of *R. sanguineus* and *Amblyomma cajennense* ticks, possibly because of the greater pressures imposed on this monoxenous and brevistrata ectoparasite for attachment and feeding, as opposed to those for the two species of heteroxenous, longirostrata ticks^34^. As mentioned, that study compared expression patterns in female ticks; this study examines for the first time the representation in other developmental stages. We verified if distribution of reads for these protein families in ticks feeding on susceptible hosts was random or biased towards a developmental stage. The results presented in Fig. 5 and Table 4 suggest that for parasitism and feeding, both nymphs and females, but not males rely on cuticle-like proteins, although they are not strongly represented. Elastin-like and glue-like proteins are found almost exclusively in males and glycine-rich proteins of the GGY family are found almost exclusively in nymphs. The more heterogeneous group of “other” glycine-rich proteins was abundantly represented in nymphs and males, but very weakly in females. In all, glycine-rich proteins were more diverse as well as abundant in nymphs, followed by males, a stage in which they were less diverse, but highly abundant. This profile may reflect the fact that males move around the host to assist females and thus need to reattach to their hosts more frequently.

**Table 4:** Representation of Transcripts Coding for Glycine-rich Proteins During Different Developmental Stages of Feeding Ticks*

Protein family	N° of reads NSGS library		N° of reads FSGS library		N° of reads MSGS library		χ^2^ test*;* *P value*
	Observed	Expected	Observed	Expected	Observed	Expected	
Cuticle-like	37	34	**37**	20	5	24	29.34; < 0.001
Cement-like glycine-rich	213	1,327	12	779	**2,839**	958	5,427.11 < 0.001
Elastin	0	14	0	8	**33**	10	74.34; < 0.001
Glue	0	33	0	19	**75**	23	168.90; < 0.001
GYG repeat-containing	4	2	0	1	1	2	N.S
GGY repeat-containing	**107	49	0	29	5	34	123.01; < 0.001
Other glycine-rich proteins	**853**	1,098	22	642	**1645**	781	1,620.62; < 0.001
Total reads in library	151.406		88.984		106.691		

*: Data are for nymphs and adults feeding on susceptible hosts; **: numbers in bold indicate that transcripts for the family are overrepresented in the indicated library.

*Representation of GRPs according to blood source*—The pie charts depicted in Fig. 5 and the data presented in Table 5 also show that in order to feed on genetically resistant Nelore hosts, *R. microplus* relies more heavily on all types of GRPs than ticks feeding on the Holsteins. Based on our present findings, one could predict, among other mechanisms, that the males are compensating the more efficient wound repair in genetically tick-resistant breeds of cattle^32,33,35^. The CDSs encoding the glycine-rich protein families (including cement, glue, elastin and others) were highly expressed in all tick libraries, except in those from females feeding on susceptible hosts (Supplemental File 1). This may occur since, in comparison with Nelore skin, inflammation is less toxic to ticks in Holstein skin^32,33,35,36^, therefore imposing fewer challenges for the cement cone of the feeding female. In addition, recent work has shown that the spacing between tongue papillae is smaller in Nelores^37^ and thus possibly more effective in removing tick larvae during self-grooming, which would explain the greater reliance of female ticks feeding on these hosts on glycine-rich proteins.

**Table 5:** Representation of transcripts coding for the main families of secreted GRPs in libraries of salivary glands from nymphs, females and males of *R. microplus* ticks feeding on genetically resistant and susceptible bovine hosts.

Library	N° of reads		Library	N° of reads		χ^2^ test *P value*	Library	N° of reads		Library	N° of reads		χ^2^ test *P value*
	Observed	Expected		Observed	Expected			Observed	Expected		Observed	Expected	
**Cuticle like proteins Cuticle like proteins Cuticle like proteins**							**Cement glycine-rich proteins**						
NSGR	**244	124	NSGS	37	157	205.88 < *0.001*	NSGR	**246**	205	NSGS	213	253	14.06 < *0.001*
FSGR	1	17	FSGS	**37**	21	24.82 < *0.001*	FSGR	**108**	53	FSGS	12	67	100.72 < *0.001*
MSGR	31	25	MSGS	5	11	3.512 N.S	MSGR	**23,955**	19,232	MSGS	2,839	7,561	4,412.03 < *0.001*
**Elastin proteins**							**Glue proteins**						
NSGR	1	0	NSGS	0	1	N.S	NSGR	0	0	NSGS	0	0	N.S
FSGR	1	0	FSGS	0	1	N.S	FSGR	0	0	FSGS	0	0	N.S
MSGR	**206**	168	MSGS	33	71	27.61 < *0.001*	MSGR	142	153	MSGS	75	64	N.S
**GYG repeat-containing proteins**							**GGY repeat-containing protein**						
NSGR	**108**	49	NSGS	4	63	122.09 < *0.001*	NSGR	**155**	116	NSGS	107	146	23.39 < *0.001*
FSGR	0	0	FSGS	0	0	N.S	FSGR	4	2	FSGS	0	1	N.S
MSGR	30	22	MSGS	1	9	9.08 *0.003*†	MSGR	7	8	MSGS	5	4	N.S
**Other glycine rich proteins**													
NSGR	**799**	729	NSGS	853	923	11.79 < *0.001*							
FSGR	**152**	77	FSGS	22	97	130.30 < *0.001*							
MSGR	**8,581**	7,245	MSGS	1645	2,981	868.94 < *0.001*							

^†^Not significant after Bonferroni correction; *N.S.: not significant; **: numbers in bold indicate that transcripts for the family are overrepresented in the indicated library.

*Representation of mucins according to developmental stage—*Regarding CDS annotated as mucins, we extracted 215 from 21,301 reads (Supplemental Files 1 and 2). Although it was the second most strongly expressed family of CDS in all libraries in relation to other protein families, the representatives of mucins were significantly (chi-square test: *P* < 0.001) more expressed in all tick developmental stages from RmR libraries (i.e., libraries derived from ticks feeding on resistant hosts; Tables 2 and 3 and Supplemental File 1). As reported herein, for most CDS encoding secreted proteins that potentially play important roles in parasitism, the mucin family is also more expressed in male ticks than in other developmental stages. We also recovered members of this family in the proteome (Supplemental File 3).

*Representation of mucins according to blood source*—The overall profile of expression of the mucin family was also affected by the source of blood supply, transcripts being significantly more abundant in ticks feeding on genetically tick-resistant Nelores (Table 2). These proteins are characterized as serine/threonine-rich proteins containing numerous potential O-galactosylation sites and are generally found in mucosal tissues. The content of tissue mast cells and other leukocytes is thought to participate in the defenses against venoms^38^, which ticks also deliver. As mentioned above, since inflammation is qualitatively different in the skin of genetically resistant bovine hosts^32,33,35,36^, with greater amounts of basophils, mucins have an important role in protecting the mouthparts and gut of blood feeding arthropods against pathogens and other insults potentially provided by leukocytes, e.g., chitinases from basophils, and hemoglobin ingested in abundance by the tick. These facts may explain why this family is abundantly transcribed and is significantly more so in ticks feeding on genetically resistant hosts.

#### Immunoglobulin-binding proteins (IGBPs)

The family of IGBPs was comprised by a total of 12 sequences (9 full length) with a predicted signal peptide (S category) and 14 additional IGBP sequences (6 full length) annotated into the H category because they did not contain a signal peptide (Supplemental File 1). Compared with male ticks fed on tick-susceptible bovine hosts, the expression of transcripts encoding for IGBPs was significantly higher when male ticks fed on tick-resistant hosts (results of chi-square analyses in columns AD-AH of Supplemental File 1). Moreover, despite previous work that identified class C IGBP as the only protein from this family to be secreted in saliva of *R. appendiculatus* ticks^39^, we found that in *R. microplus*, IGBPs of classes A, B and C present a predicted signal peptide and, therefore, might be secreted in saliva (Supplemental File 1, SigP Result in column AI). Indeed, all three classes of IGBPs were identified in the proteome (Supplemental File 3). Moreover, 26 CDSs for IGBPs were found in the transcriptome. IGBPs have been found in all male hard ticks examined to date, including in *Ixodes scapularis*, which frequently mates off its hosts. The exception is *Hyalomma dromedarii*^40^, where transcripts for IGBPs were not found in the salivary gland transcriptomes from males and females feeding on camels and a partial sequence was obtained for a single type of IGBP in the corresponding proteome. Camelids of the Saharan regions are the natural hosts of *H. dromedarii*^41^, mammals which produce mainly single chain antibodies, instead of conventional antibodies. Towards understanding why males of this species do not produce an array of IGBPs it will be interesting to examine if IGBPs from other species of ticks bind to single chain antibodies from camelids.

It has been reported that most male ticks produce IGBPs to protect themselves and the females from their host's antibody-mediated immunity^42^. IGBPs are an escape mechanism common to pathogens and parasites against which antibodies constitute important effector mechanisms^43^ and certain host IgG allotypes are bound less efficiently by them^44^ and result in a more favorable outcome for the host. In the same manner, certain bovine allo-haplotypes of IgG2 are associated with phenotypes of infestations with *R. microplus*^45^, which underscores the importance of this mediator of escape mechanisms. Although transcripts and proteins of this family were highly expressed and produced almost exclusively by male ticks, we also recovered these salivary proteins in other developmental stages as nymphs and engorged females. The presence of IGBPs in the saliva of nymphs may be explained by the molting of nymphs to adult males, whereas the presence of those proteins in females may be due the mixing of between male and female saliva in the blood pool where they jointly feed. Indeed, Wang and Nuttall detected IGBPs in the haemolymph of female ticks^42^. Levels of tick saliva-specific IgG antibodies are down modulated in heavily infested, genetically tick-susceptible bovines when compared to levels seen in genetically tick-resistant hosts managed in the same pasture and this may be due to the action of tick IGBPs^17^.

#### Antimicrobial peptides (AMPs) and proteins with trypsin inhibitor-like (TIL) domain-containing proteins

Other families of CDS that were highly expressed in nymphs and males feeding on susceptible hosts were those that encode proteins with antimicrobial properties, i.e., the AMPs and TIL domain-containing proteins (Figs. 3b and 4, and Tables 2 and 4 and Supplemental File 1). The family of AMPs is composed by defensins, longicornsins, hebreains, ricinusins, microplusins, inhibitors of neutrophil elastases and lysozymes (Supplemental File 1). Members of this diverse family were also recovered in the proteome (Fig. 3b, asterisks on the right of the heatmap). One member of this family has previously been identified and characterized in hemolymph from *R. microplus* as microplusin^46^. Microplusin is a multifunctional protein having antimicrobial actions against several pathogens. AMPs may regulate the microbiota of the tick’s salivary glands^47^ and of the host’s skin during attachment, consequently affecting local immunity^48,49^ in favor of the parasite. It is noteworthy that nymphs and males feeding on susceptible hosts expressed significantly more transcripts for AMPs than females; females will eventually drop to the ground, an environment burdened with microbial threats for this developmental stage as well as for their clutches of eggs. The expression profile of salivary glands from detached females was not examined in the present study, but this stage may begin to transcribe more AMPs when they are ovipositing on the ground. Esteves and colleagues found that expression of a defensin did increase within the period of blood feeding in salivary glands of females of *Amblyomma sculptum*^50^. However, alternatively, they may acquire the necessary AMPs from the males during their joint blood meal. Indeed, Xavier and colleagues^51^ recently examined the transcriptome and proteome of the Gené’s organ, which secretes a protective wax with which females coat their eggs; in spite of the fact that antimicrobials have been described in this wax, the transcriptome presented relatively few corresponding transcripts clustered into 5 CDS versus the 47 shared between salivary glands of nymphs and males feeding on susceptible bovines observed in the present study. Thus, in addition to the antimicrobial activities provided by the free fatty acids produced by the Gené’s organ, the AMPs acquired from the male tick may also protect females from infections after they have dropped from the host. TIL domain-containing proteins were also abundantly expressed in males, however, they were not recovered in the corresponding proteomes; they are also thought to have antimicrobial properties. This role is suggested by data on BmSI-7, one of the TIL inhibitors described in *R. microplus*. This protein inhibits subtilisin A and Pr1 proteases of entomopathogenic fungi^52^.

**Figure 6:**
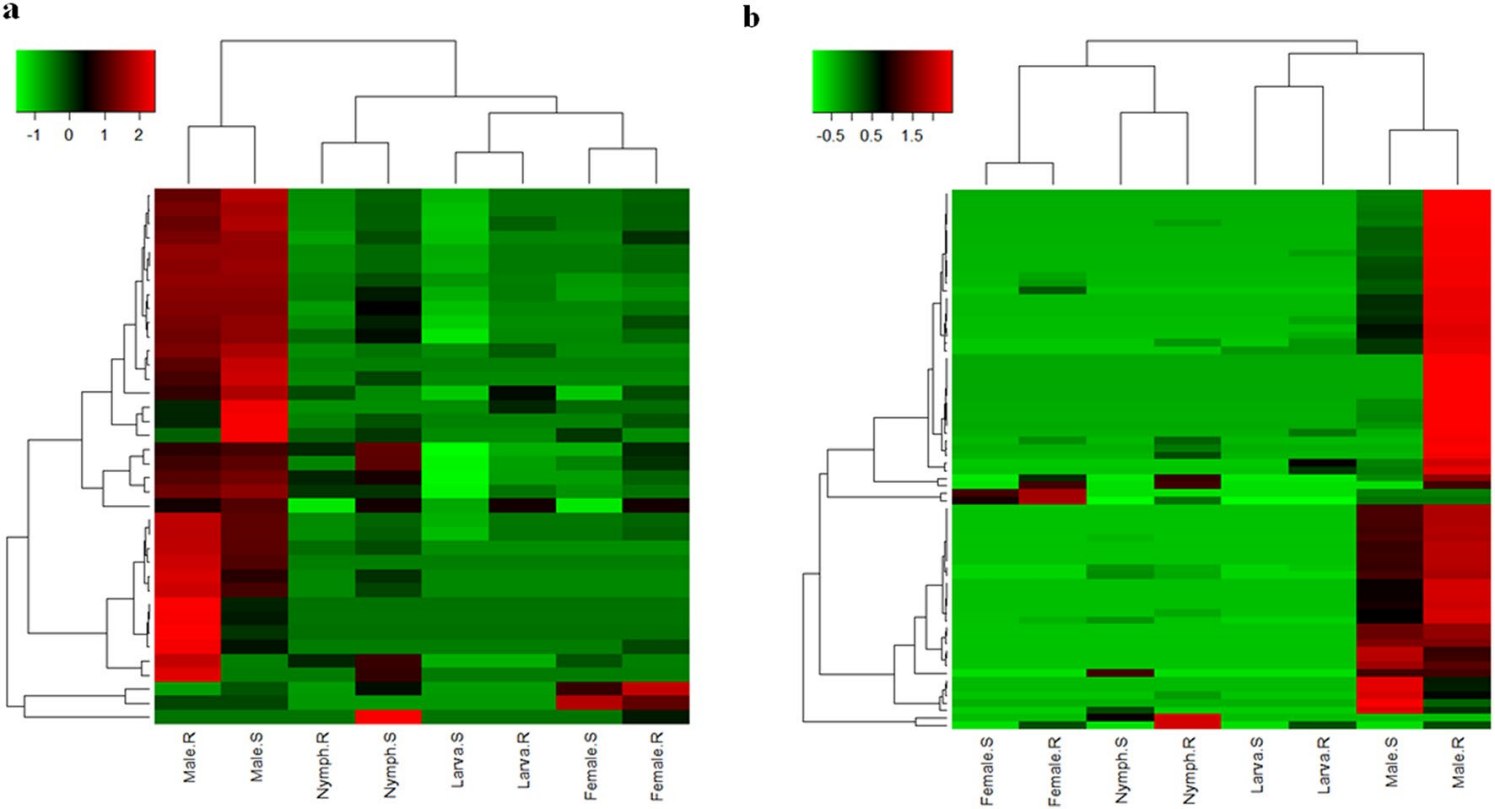
Transcriptional profiles of CDSs encoding proteins with potential antimicrobial properties. Heatmaps are clusterized by dissimilarity and are based on the expression of CDSs encoding defensins, longicorsins, lysozymes and microplusins (**a**) and TIL domain-containing inhibitors (**b**). Color scale: green, black and red, less to more expressed, respectively. The letters R and S indicate stages feeding on resistant and susceptible hosts, respectively.

Figure 6 depicts the heatmaps for the expression of the CDSs of these AMP and TIL-domain containing families; they indicate that male ticks abundantly express genes encoding proteins with potential antimicrobial properties. Moreover, the figures show that the source of blood affected the level of transcription of certain individual CDSs within the families. Finally, Table 6 shows the distribution of CDDs encoding AMPs and TIL inhibitors according to life stages and source of blood. Towards understanding the functions of these distinct CDSs, it will be interesting to examine if females that have fed on resistant or susceptible bovines present different levels of susceptibility to enthomopathogens when they are ovipositing in the ground.

**Table 6:** Representation of transcripts coding for the main families of secreted AMPS and TIL-domain-containing proteins in libraries of salivary glands from nymphs, females and males of *R. microplus* ticks feeding on genetically resistant and susceptible bovine hosts.

Origin of Blood Meal	Nelores								Holsteins							
Origin of library	Larvae**		Salivary glands from						Larvae**		Salivary glands from					
			♂		♀		Nymphs				♂		♀		Nymphs	
Family of AMP	N^o^ of Transcripts															
	Observed	Expected	Observed	Expected	Observed	Expected	Observed	Expected	Observed	Expected	Observed	Expected	Observed	Expected	Observed	Expected
**Defensins**^**†**^** (22 CDDs)**	***23***^**††**^	11.96	***429***	536.08	28	27.33	***17***	39.71	***8***	19.03	***332***	224.917	34	34.67	***73***	50.29
**CDD Rm-tb2-7,189**	2	–	***65***	98.65	0	–	0	–	0	–	***75***	41.3492	0	–	1	–
**CDD Rm-nr-89218**	1	–	***213***	179.03	1	–	1	–	0	–	***41***	74.9717	0	–	3	–
**CDD Rm-nr-113771**	0		13		1		11		0		**4**		3		43	
**Longicornsins**^**†**^** (4 CDDs)**	0	–	***54***	88.78	0	–	0	–	0	–	***72***	37.2147	0	–	0	–
**CDD Rm-nr-36765**	0		**14**		0		0		0		**16**		0		0	
Microplusin family*^†^ **(9 CDDs)**	6	–	***281***	359.31	***36***	16.31	***37***	53.83	4	–	***229***	150.692	***1***	20.69	***85***	68.17
CDD Rm-nr-54461	5	–	***266***	335.37	0	–	***4***	8.38	3	–	***210***	140.634	0	–	***15***^***#***^	10.62
**Neutrophil elastase inhibitors with AMP domain**^**†**^ **(5 CDDs)**	0	–	10	9.87	***116***	53.82	***139***	174.25	0	–	4	4.13	***6***	68.18	***256***	220.75
**CDD Rm-nr-117512**	0		6	5.60	2	–	***139***	172.04	0	–	2	2.40	0	–	***251***	217.96
Other intermediate proteins*^,†^ **(13 CDDs)**	***136***	53.33	***50***^***#***^	42.99	25	12.34	27	23.83	***2***	84.67	***11***	18.01	3	15.66	27	30.17
Rm-nr-32425	***103***	41.33	0	–	0	–	0	–	***1***	65.66	0	–	0	–	0	–
Lysozymes*^,†^ (3 CDDs)	1	–	24	21.85	0	–	0	–	0	–	7	9.152	0	–	0	–
TIL-domain containing AMPs*^,†^** (72 CDDs)**	13		1,145		50		29		0		973		13		37	
Total No reads for AMPs	***166***	69.57	***848***	1,058.35	***205***	109.90	***220***	291.50	***14***	110.42	***655***	444.65	***44***	139.10	***441***	369.50
Total N^o^ reads/library	54,115		254,657		70,130		119,574		86,135		106,691		88,984		151,406	

*: putative AMP by similarity with AMPs tested for antimicrobial activity; **: ecloded from eggs oviposited by females fed on the indicated hosts; ^†^Total number of transcripts/library in all contigs annotated for a similar putative biological function derived from a developmental stage;^††^Numbers in bold indicate a significantly different proportion of transcripts in the same developmental stage feeding on different hosts (*P* < 0.05, χ^2^ test); ^#^Not significant after Bonferroni correction of χ^2^ test.

#### Lipocalins

We recovered 294 CDS deduced from 10,524 reads presenting significant similarity to the lipocalin family, such as P27 protein and serotonin- and histamine-binding proteins (SHBP), among other lipocalins. Of these, 143 CDS are full length and presented a predicted signal peptide, indicative of secretion. Another 56 full length sequences have no predicted signal peptide. The biological significance for a family having such high representation warrants investigation. A finding in this study that can shed light on the significance of the lipocalin family for the tick-host interface is the fact that it was also one of the few families that was represented with greater diversity and abundance in females feeding on susceptible bovine hosts instead of nymphs and males *ibid* (more than 7,300 transcripts clustered into 289 CDSs). Lipocalins bind to and transport small hydrophobic molecules such as steroids, bilins, retinoids and lipids^53^. They are present in all species of soft and hard ticks studied to date^54^ and perform anticlotting^55^ and anticomplement^56^ activities, and regulate hemostasis and inflammation by scavenging biogenic amines^57^, ADP^58^, leukotrienes and thromboxane A_2_^59,60^. Furthermore, histamine and serotonin are important mediators of inflammation and hemostasis and seem to be critical in regulating tick engorgement and transmission of pathogens^61^. Resistance to *R. microplus* has been shown to correlate with the amount of histamine in cattle skin^62^, but our results do not reflect a complementarity between amounts of skin histamine and transcription of lipocalins: among the 294 CDS encoding putative lipocalins, only one, Rm-nr-14812, was overrepresented in salivary glands of ticks feeding on Nelores. This also implies that, besides binding histamine, lipocalins must exert other functions in parasitism by the tick. In addition, the great diversity of CDS encoding lipocalins may be because R*. microplus* remains attached to its hosts for three weeks and antigenic variation might be provided by selectively expressing different members of this protein family.

### Highlights of the new catalogue: insights into the biology of *R. microplus*, a monoxene, slow feeding tick

Towards understanding the mechanisms behind adaptations of *R. microplus* ticks to their bovine hosts, the present study assembled a comprehensive catalogue of CDSs encoding proteins of *R. microplus* ticks and a proteome generated with MudPIT of the corresponding biological materials employed to generate the CDSs. Briefly, compared with previous studies that addressed the functional genomics and the proteome of saliva from *R. microplus* ticks, this catalogue possesses a greater number of sequences, as well as a greater number of complete sequences. In addition, it addresses the dynamics of expression of salivary constituents according to all developmental stages *R.* microplus, except for feeding larvae. Finally, it also addresses the effect of the sources of blood from bovine hosts of different genetic backgrounds that result in contrasting phenotypes of infestations, with significant differences in tick loads.

When examining these catalogues for insights about the biology of *R. microplus*, we considered several characteristics. The number of hosts visited by ixodid ticks during the parasitic stage of their life cycle and the period of the blood meal require several adaptations: ixodid ticks are slow feeders and they die when they are unable to find a host. In this regard, *R. microplus* is a monoxene, or one host tick and its parasitic, blood-feeding stage lasts around 21 days. According to Balashov^63^ and to Hoogstraal and Kim^64^ there was a transition from a three host to two and one host cycles in Hyalomma and in Rhipicephalinae species of ticks. The biological characteristic of having a single host is thought to be an adaptation of this immobile, hematophagous parasite to large nomadic mammals since monoxenous ticks are better adapted to open environments inhabited by large, grazing ungulates. The ability to molt on the vertebrate host reduces the number of necessary encounters and thus increases chances for tick survival. Feeding continuously for approximately 3 weeks entails the ability to sequentially recruit molecules to counter the host’s defenses that, in the case of responses to a monoxene tick, will range from innate to acquired immune responses since several tick salivary proteins are common to all morphs of the parasitic stage. Finally, *R. microplus* is thought to have co-evolved with indicine cattle on the Indian subcontinent^30,31^; breeds of this subspecies of bovines carry significantly lower loads of ticks than taurine breeds of cattle, which can suffer debilitating infestations. Studies have shown that these contrasting, highly heritable phenotypes of infestations are, in part, a consequence of the composition of host responses to tick bites^32,33^. Herein, we sought to examine if there are corresponding changes in the parasite’s sialome. While it would be interesting to also contextualize the sialotranscriptome described in the present study in terms of the evolution of monoxene versus heteroxene and monotropic versus telotropic ticks, at this time there are no sialotranscriptomes adequate for this purpose. This said, we will, however, compare the sialotranscriptome of this study with two of male ticks that are currently available for heteroxene species.

#### Composition of the sialome changes with the developmental stage of *R. microplus*

The hierarchical clustering of expression profiles in different libraries provided several insights about the biology of parasitism of the cattle tick. Firstly, it showed that in unfed larvae (the libraries for which were constructed with whole morphs), almost all families of proteins, including those of H and S categories, had intermediate to low or absent expression, except the families of glycine-rich proteins, including cements, mucins, the 10 KDa protein family and several unknown conserved proteins. In other words, unfed larvae are equipped to begin attaching to their hosts. The 10 KDa protein family presents similarities to tick and worm peptides rich in GY repeats. The abundance of Tyr residues may provide for cross linking of these peptides upon phenol oxidase activity. In arthropods, these enzymes participate in sclerotizing the proteins in a flexible exoskeleton^65^. In addition, peptides rich in GY repeats have antimicrobial activity in worms^66^. Interestingly, many CDSs encoding proteins of unknown functions were expressed exclusively in unfed larvae (Supplemental File 1).

We also observed that during the nymphal stage, the CDS encoding protein families that were weakly expressed in the larval stage begin to increase their transcription, such CDS encoding proteins involved in energy metabolism, transcription machinery, protein synthesis machinery, protein modification machinery, signal transduction, lipocalins, AMPs, Kunitz domain-containing protease inhibitors, among others. As noted above, lipocalins were abundantly expressed in nymphs and adults, but much more strongly in the female.

Another insight revealed by hierarchical clustering is the conspicuously greater diversity and abundance of transcripts of CDSs encoding secreted salivary proteins related to hematophagy and parasitism in the male’s salivary glands, a finding that was confirmed with statistical tests. In other words, the male tick is indeed crucial to assist the female in hematophagy, firstly by maintaining its efficient mobility and attachment and then by maintaining the hemorrhagic pool and by neutralizing the hosts’ defense mechanisms. In addition to the information generated on IGBP and GRPs and mucins, discussed above, this study also showed that the male tick’s saliva contains a protein similar to longistatin with a signal peptide (indicative of secretion). Longistatins have been characterized as plasminogen activators and anticoagulants, two important functions for formation of the blood pool^67^; in addition, they inhibit inflammation by binding to receptors of advanced glycation end products^68^.

Male salivary glands also uniquely assist fertilization, related to their peculiar mode of reproduction^69–71^. Indeed, a sialotranscriptome of male and female *R. pulchellus* showed many transcripts uniquely associated with male ticks, several of which are from families commonly found in insect male accessory glands and vertebrate prostate glands and that were at least one hundred times more expressed in salivary glands of males compared with females^72^. Similarly, in the present study we found 87 transcripts in the present *R. microplus* sialotranscriptome that significantly accumulated more than 100 times in male-derived reads than female-derived reads (Supplemental file 1, column Z of the spreadsheet), and additional 781 transcripts that were significantly accumulated more than 10 times in male-derived reads. Sixty percent of these transcripts code for proteins of the secreted class, including serine proteases, longistatin, male IgG binding lipocalins, metalloproteases, serpins, cystatins, TIL domain containing antimicrobial peptides, etc. Taken together, these results from *R. pulchellus* and *R. microplus* sialotranscriptomes indicate the presence of a large number of male-specific salivary gland transcripts that might have a direct role in tick reproduction in addition to functions of hematophagy and parasitism per se.

#### Composition of the sialome is complementary with host defenses

Another important result of this study was the finding that the profiles of the transcriptome and composition of the proteome are strongly affected by the genetic background of the bovine host on which cattle ticks feed. One of the consequences of this effect is that there is a delay of expression profiles in ticks feeding on resistant bovines: the expression profile seen in nymphal salivary glands feeding on susceptible bovines is presented in these ticks only when they reach the adult, female stage. The relative representations of transcripts for lipocalins, metalloproteases and protease inhibitors containing a Kunitz domain were high in nymphs feeding on Holsteins and in females feeding on Nelores; conversely, the relative representations of transcripts for mucins and proteins of the 10 KDa family were low in nymphs feeding on Holsteins and in females feeding on Nelores. These findings suggest that, for example, insufficient Kunitz inhibitors in saliva may result in less nymphs molting to females on Nelores. The source of the blood significantly affected transcription of the lipocalin family, being down modulated in ticks feeding on resistant hosts. In addition, the expression profiles of nymphs feeding on susceptible hosts and females feeding on resistant hosts are similar, indicating that ticks that confront the host responses more precociously with salivary proteins such as Kunitz-domain containing proteins, metalloproteases, Antigen 5-type antioxidant proteins and evasins will be more successful at feeding and reproductive efficiency.

One noteworthy finding concerns the relatively very high level of expression of the 10 KDa protein family in nymphs feeding on Nelore hosts compared to those feeding on Holsteins. As noted above, these proteins are rich in GY repeats and participate in sclerotizing the proteins in flexible exoskeletons and have antimicrobial activity. This “sialopathology”, triggered by the Nelore’s immunity and whereby nymphs are producing too much GY protein, may result in excessive sclerotization (detrimental for engorging and molting) and in a dysbiosis of nymphs.

Another effect of the host genotype was upon the expression profiles of the male tick: compared to profiles presented by the male feeding on Holsteins, transcripts encoding proteins of the mucin, glycine-rich and 10 KDa families dominated the profile of males feeding on Nelores, while transcription of CDS encoding the remaining dominant families was down-regulated. Males feeding on susceptible hosts presented the most functionally diverse range of CDS encoding secreted salivary proteins. Again, this finding points to the crucial role played by the male in parasitism. However, the greater abundance of transcripts encoding several families of proteins in *R. microplus* fed on tick-resistant hosts (mucins, glycine-rich proteins and IGBPs) may also be explained by the hypothesis that high levels of host immunity generate a “super” *R. microplus* tick, with more ability to modulate the responses generated by resistant hosts until its entire life cycle is complete, even at the cost of apparent lower reproductive success, i.e., less eggs,viable larvae and ticks reaching the adult stage.

Rodriguez-Valle and colleagues were the first to show that composition of the sialome is complementary with host defenses by examining gene expression profiles of *R. australis* ticks feeding on Brahman (an indicine breed) and Holstein–Friesian bovines^73^. That study examined whole females and larvae that were exposed to, but not allowed to feed on these hosts; differently from the present study, it did not examine nymphs and males and did not employ salivary glands. In addition, it evaluated gene expression with a microarray constructed with 13,643 members of BmiGI Version 2 that represents pooled tissues of whole ticks and tissues undergoing different stressors^26^. Nevertheless, in terms of expression mediators of parasitism, when that study and the present are compared, some findings merit comment. Firstly, Rodriguez-Valle and colleagues did not examine male ticks and their tissues, however they found that expression of IGBPs was relatively higher in whole female ticks feeding on Brahman hosts. In the sialotranscriptome generated in the present study, only 5 out of over 2,000 transcripts for IGBPs were found in salivary glands from females. Those authors also found that expression of a lipocalin and of a histamine-binding protein were upregulated in whole female ticks feeding on Brahmans. Out of 295 CDDs encoding lipocalins and histamine-binding proteins, the present study only observed a similar effect of indicine hosts on feeding ticks in less than 30 CDDs and they were all relatively upregulated in salivary glands from males and nymphs feeding on indicines (Supplemental File 1). Finally, those authors showed that expression of proteinases was upregulated in females feeding on Brahmans, and the present study observed the contrary (Table 2 and Supplemental File 1). The discrepancies between these studies may be because Rodriguez-Valle and colleagues employed whole females and a microarray with a limited representation of what is expressed in salivary glands. It should be considered, additionally, that recent transcriptomic studies indicate a temporal change in the sialome expression within the same tick stage, a phenomenon described as “sialome switching”^74,75^, which can occur within 24 h intervals^76^. Accordingly, comparisons between studies could be difficult unless the tick samples are collected and compared at the same conditions. We also point out the present sialome was generated with ticks sampled starting from the 11th until the 21st day after infesting larvae were released on the bovine hosts and examined for features of each instar before pooling the samples as nymphs, females or males. Thus, sialome switching was covered, but for the reasons just stated, certain comparisons cannot be made between studies.

Maldonado-Ruiz and colleagues recently showed that the antigen content of salivary glands from *Amblyomma americanum* ticks raised in a colony or collected from the wild (and this feeding on different hosts) presented with different profiles of reactivity in ELISA and immunoblots with serum antibodies from humans exposed to this species of tick^77^. Similar results have been described by Garcia and colleagues^16^. Likewise, Tirloni and colleagues^78^ showed that both *I. scapularis* and *A. americanum* female ticks differentially express saliva proteins when stimulated to start feeding on rabbits, humans or dogs. Finally, Narasimhan and colleagues^79^ also showed that salivary glands of *I. scapularis* nymphs when fed to repletion on guinea pigs, which are resistant to infestations with this species of tick, or mice, which are permissive, also present differential gene expression profiles that also correlate with profiles of inflammation and immunity. These investigators also showed that expression of most CDDs encoding histamine-binding proteins was more abundant in ticks feeding on mice. These finding concur with ours about expression of this family of proteins in ticks feeding on indicine or taurine hosts. In all, these findings agree with those of this study, which shows that the source of blood affects the expression profile in salivary glands including for putatively secreted proteins. Again, we should warn that any reported differences could be due to differences in the sialome switching among the different treatments.

#### Comparisons between the sialotranscriptomes of males and females of a one-host and of two three-host ticks of the genus Rhipicephalus

To the best of our knowledge, the cattle tick *R. microplus* is the only monoxene species of the genus *Rhipicephalus* for which a comprehensive sialotranscriptome is now available. In order to verify if there are some salivary components that confer *R. microplus* the ability to maintain this biological feature, we took the opportunity to examine its sialotranscriptome comparatively with that of *R. pulchellus* generated by Tan and colleagues^72^ with salivary glands of females and males fed on rabbits and that of *R. zambeziensis* generated by De Castro and colleagues^80^ also with salivary glands of females and males fed on a taurine breed of cattle. *R. pulchellus* is a three-host tick that infests a wide range of hosts in Africa, including humans, livestock animals such as cattle and goats, and wild animals such zebras, giraffes, elands and hares^5^; *R. zambeziensis*, which occurs in the drier areas of the African continent, is a three–host tick and its adults feed on cattle, impala, greater kudus and lions, while immature stages can feed on hares^5^.

Interestingly, gender-specific levels of expression in the *R. pulchellus* sialotranscriptome for CDDs encoding evasins, Dap36 immunosupressants, Antigen 5 antioxidants, glycine-rich proteins, mucins, AMPs, IGBPs and lipocalins followed the same profile as that of the sialotranscriptome of *R. microplus*. The exception was the level of expression of metalloproteases, where males of *R. microplus* expressed almost six times more transcripts of these proteins than females, opposite profiles being observed in *R. pulchellus*. Regarding the combined time-point profiles of expression of transcripts encoding these proteins in males and females of *R. zambeziensis*, they were also similar to those seen in *R. microplus* adults with the exception again of metalloproteases, but also of evasins, 8.9 kDa protein family and mucins, where females presented greater representation of these transcripts than males. We have already pointed out above the possible explanation for the discrepancy between levels of expression of IGBP in the sialotranscriptome generated in the present study and in that of males of the two- or three-host tick *H. dromedarii* that can infest cattle as well as camelids^40,41^.

At present it is not possible to ascribe a role for secreted salivary protein families in determining the number of hosts different species of ticks have in their life cycles. For this exercise to yield useful information for understanding tick biology, more information is necessary about these species’ preferred hosts and the profiles of local inflammatory responses that they mount. Mating strategies of these ticks must also be described in more detail in order to understand each species needs in mate guarding.

### The contribution of the new sialotranscriptome of *R. microplus* ticks for development of a vaccine to control cattle ticks

Among the previous studies already mentioned herein aiming to identify new targets to compose a vaccine that controls tick loads in cattle, one identified 791 proteins using a combination of DNA microarrays, in silico prediction and antigenicity studies^81^. The expression profiles in midguts of feeding adult females, salivary glands and ovarian tissues were also evaluated employing microarray and ESTs from females of *R. microplus*^82^. From 13,456 transcripts this study assembled 588 genes that were expressed in all three tissues and that included secreted anti-hemostatics, defense proteins, proteases, enzymes, transporters and proteins involved in digestion, DNA replication and cell-cycle control for oogenesis in the ovaries. The same research group examined differential gene expression using comparative microarray analyses of midgut from adult females of *R. microplus* and *R. decoloratus* ticks to discover cross-reactive antigens between species of ticks^83^. The analyses showed that 2,476 genes were shared between these *Rhipicephalus* species; in addition, 1,084 transcripts were more abundantly expressed in *R. microplus,* but transcripts associated with lipid transport and metabolism were equally expressed in both ticks. These studies also lack information about how salivary constituents are used during the tick’s life cycle and in infestations in hosts with different levels of resistance. Other studies show how the infection of the host with a tick-borne hemoparasite affects the transcriptional profile of *R. microplus* ticks. Several transcripts from ovary and gut of *R. microplus* had their expression increased in ticks feeding on *B. bovis*-infected hosts, in comparison with ticks fed on uninfected hosts^84,85^. The expression profiles revealed in the present study underscore the importance of salivary proteins of nymphs and males as vaccine targets. Systematic approaches to study gene expression profiles of salivary glands from pathogen-infected *R. microplus* ticks are now necessary in order to discover proteins that may be involved in vectorial competence and thus also representing useful antigens.

## Conclusions

The transcriptional profile and protein content of salivary glands of *R. microplus* is affected by both the developmental stage of the tick and the source of blood. Figure 4 is representative of the main findings in this study. Levels of expression for CDSs encoding chemokine-binding evasins and metalloproteases are consistent along the developmental stages of nymphs to females and males. This suggests that even if males assist the females’ blood meal, this latter morph must rely on its own mechanism to avoid recruitment of leukocytes and to maintain peptidase activities. On the other hand, expression of CDS encoding glycine-rich proteins (GRPs) and mucins increase greatly in males relative to nymphs, while females weakly express CDS encoding these families of proteins. This suggests that the males’ GRPs and mucins are guarding the female or that males have greater demands for GRPs and mucins because it moves about the host assisting females. Expression of CDS encoding Kunitz domain-containing proteins increase greatly in females relative to nymphs, while males express the lowest amounts of CDS encoding these families of proteins. This suggests that increasingly larger amounts of proteases that need to be inhibited are being ingested in the blood meal while nymphs morph into females. The largest amounts of transcripts were for CDSs encoding lipocalins, in nymphs and even more so in females; males weakly expressed this family. This suggests that only nymphs and females must scavenge small hydrophobic molecules such as steroids, biogenic amines and lipid mediators. Transcripts for CDSs encoding serine proteases were more represented in males. Feeding on resistant hosts greatly increased the levels of expression of CDSs encoding GRPS and mucins and the 10KDa, GY-rich protein family; this latter family was very weakly expressed by all stages of ticks when feeding on susceptible hosts. Proteins of this family have been shown to present antimicrobial activity. Why this family needs to be recruited when tick feed on resistant hosts needs to be elucidated.

## Methods

### Ticks, hosts and sample preparation

All materials employed to generate the transcriptome and proteome were derived from ticks obtained from a colony maintained by the University of São Paulo. This colony is located at latitude and longitude 21.17201743031726 and − 47.854628700524934, respectively, and uses bovines of the tick-susceptible Holstein breed of *Bos taurus*. To begin the experiment fully engorged females were removed from the hosts, washed in sterile distilled water and incubated at 27–28 °C with 80–90% relative humidity until oviposition, approximately 2 weeks. The resulting egg masses were pooled and separated into batches of 500 μg, placed in in 20 ml syringes with the ends containing the needle sleeves cut off and stoppered on each end with cotton plugs or the plunger. The eggs were further incubated under the same conditions until 3 weeks after the larvae ecloded; this procedure generates approximately 5,000 infesting larvae in each syringe. These larvae were released on the backs of tick-susceptible (*Bos taurus,* Holstein breed, N = 4, 5,000 larvae per host; S) or resistant (*Bos indicus,* Nelore breed, N = 12, 10,000 larvae per host; R) hosts by removing the cotton stopper and pushing the plunger into the syringe. Hosts were always 30 days past the window for residual effects of any past applications of acaricides.

From the 11th until the 14th day after releasing the larvae, and the 17th until the 21st day *ibid*, nymphs and adults (males and females) were collected on each group of hosts, R and S. After removal from hosts the salivary glands (SG) were dissected within one hour using a LEICA MZ 9.5 magnifying glass (Meyer Instruments, Inc., Houston, TX, USA) and sterile dissection materials and immediately stored in RNALater (Ambion Inc., Austin, TX, USA) and frozen at − 80 °C for extraction of RNA, or solubilization buffer (7 M urea, 2 M thiourea, 0.2% SDS, 1% DTT, 2% Triton X-100, Protease and Nuclease Inhibitor cocktail) for proteomic analysis by MudPIT technology. Ticks were harvested during the summer and early autumn. Females removed from R and S hosts were incubated to generate the unfed larvae using the same procedure described above; three weeks after ecloding, 1 mg of larvae derived these females (UFLS and UFLR, respectively) were immersed in RNALater or solubilization buffer and frozen.

The transcriptome was generated with the following pooled materials and respective amounts: unfed larvae derived from 1 mg of egg masses oviposited by females fed on S (UFLS) and R (UFLR); 280 and 192 pooled pairs of SG from nymphs feeding on S (NSGS) and R (NSGR) hosts, respectively; 157 and 201 pooled pairs of SGs from males feeding on S (MSGS) and R (MSGR) hosts, respectively; 244 and 119 pooled pairs of SGs from females feeding on S (FSGS) and R (FSGR) hosts, respectively. The proteome was derived from materials generated with a similar number of ticks. In addition, semi-engorged female ticks were also employed to obtain the saliva samples (SalivaS, from female ticks fed susceptible and SalivaR, fed on resistant hosts) for proteomic analyses. To induce salivation, a 0.2% dopamine solution was injected into the hemocoel of tick and the saliva was collected in solubilization buffer using a sterile pipette tip. All tick samples were stored at − 80 °C until use.

### RNA isolation, library preparation, pyrosequencing and bioinformatic analysis

The workflow employed to RNA isolation, library preparation, pyrosequencing and bioinformatic analyses, including the hierarchical clustering analyses, multiple sequence alignments, venn diagram and phylogenetic analyses were performed exactly as previously described by Garcia and colleagues^14^. Non-normalized library preparations for GS FLX titanium (Roche/454 Life Sciences, Branford, CT, USA) sequencing were developed in the High-Throughput Sequencing and Genotyping Unit of the Roy J. Carver Biotechnology Center of the University of Illinois at Urbana-Champaign, based on standard methods used in GS FLX sequencing. After obtaining the sequencing output, numerous bioinformatic and manual analyses were performed as previously described^86^, and the functionally annotated sialotranscriptome of *R. microplus* is presented in hyperlinked Excel spreadsheets and designated as Additional file 1 (the weblinks are best visualized with Internet Explorer, Google Chrome and Mozilla Firefox browsers) and functionally described in Additional file 2. The statistical analysis used to observe the differential transcript abundance between *R. microplus* libraries was the chi-square (χ^2^) test. We employed the number of reads from each coding sequences of different libraries and the results were considered statistically significant if *P* < 0.05. Bonferroni correction was used for testing significance of multiple comparisons between dominant protein families. Automatic annotation was based on searches by BLAST algorithms against public protein databases and customized databases created using previously identified tick proteins. This exercise was followed by manual annotation of CDS according to their putative function through detailed analysis of the BLAST searches as previously reported^23^ (Supplemental Files 1 and 2).

### Identification of *Rhipicephalus microplus* proteins by multidimensional protein identification technology (MudPIT)

The extracts of UFL, NSG, MSG, FSG and Saliva obtained from *R. microplus* fed on tick-resistant Nelore or tick-susceptible Holstein hosts, totaling 10 extracts that were employed to provide the *R. microplus* sialoproteomes. The methods employed in proteomic analysis were performed according contracted services at Chemical and Proteomic Mass Spectrometry Core Facility, Virginia Commonwealth University. Briefly, 10 μL of each solution was removed for processing and diluted to 100 μL with 100 mM ammonium bicarbonate. Protein concentration of SGs and saliva ranged from 1.6 to 4.5 μg/μL and were determined with a standard Bradford assay. These samples were reduced with 5 µL of 10 mM dithiothreitol in 0.1 M ammonium bicarbonate at room temperature for 30 min. Then they were alkylated with 5 µL 50 mM iodoacetamide in 0.1 M ammonium bicarbonate at room temperature for 30 min. The samples were digested with 1 ug trypsin overnight and then quenched with 5% (v:v) glacial acetic acid. Each sample was desalted off-line prior to mass spec analysis. The LC–MS system consisted of a Thermo Electron LTQ-Orbitrap hybrid mass spectrometer system (Thermo Scientific Pierce, Waltham, Massachusetts, USA) with a nanospray ion source interfaced to a Waters NanoAcquity C18 reversed-phase capillary column (Waters corporation, Milford, Massachusetts, USA). The C18 trap column on the system was fronted with an SCX trap column. About 2–5 µg of the final solution was injected and the peptides which were not trapped on the SCX column were eluted from the column by an acetonitrile/0.1 M formic acid gradient at a flow rate of 0.4 µL/min over 60 min. After this flow through fraction, eight ion exchange fractions were eluted onto the C18 column, using aliquots of increasing concentration of ammonium acetate. Each ion exchange fraction was analyzed by an acetonitrile/0.1 M formic acid gradient at a flow rate of 0.4 µL/min over 60 min. The nanospray ion source was operated at 3.5 kV. The digest was analyzed using the double play capability of the instrument acquiring full scan mass spectra to determine peptide molecular weights and product ion spectra to determine amino acid sequence in sequential scans. This mode of analysis produces approximately 100,000 CAD spectra per ion exchange fraction of ions ranging in abundance over several orders of magnitude. Not all CAD spectra are derived from peptides. The data were analyzed by database searching using the Sequest search algorithm (Thermo Fisher Scientific, San Jose, CA, USA; version 1.2.0.206) against custom *Rhipicephalus microplus* tick database (the sialotranscriptomes from *R. microplus* described here; Additional File 1; and deposited as BioProject ID PRJNA329522) assuming the digestion enzyme trypsin. Sequest was searched with a fragment ion mass tolerance of 0.80 Da and a parent ion tolerance of 15 PPM. Iodoacetamide derivative of cysteine was specified in Sequest as a fixed modification. Oxidation of methionine was specified in Sequest as a variable modification. The Scaffold software (version Scaffold_3.4.3, Proteome Software Inc., Portland, Oregon, USA) was used to validate MS/MS based peptide and protein identifications. The criteria for protein identification were the following: Peptide identifications were accepted if they could be established at greater than 95,0% probability as specified by the Peptide Prophet algorithm^87^. Protein identifications were accepted if they could be established at greater than 99.0% probability and contained at least 2 identified peptides. Protein probabilities were assigned by the Protein Prophet algorithm. Proteins that contained similar peptides and could not be differentiated based on MS/MS analysis alone were grouped to satisfy the principles of parsimony. The peptides and proteins identified in *R. microplus* proteomes using custom *R. microplus* database are shown in Additional File 3.

### Accession numbers

The sequences from *R. microplus* were deposited at GenBank (NCBI) as BioProject ID PRJNA329522 containing eight biosamples (SAMN05439577, SAMN05439580, SAMN05439579, SAMN05439578, SAMN05439573, SAMN05439576, SAMN05439575 and SAMN05439574), named as: (1) Unfed larvae from tick fed on resistant bovines; (2) Nymph salivary gland from tick fed on resistant bovines 11–14d post attachment; (3) Female salivary gland from tick fed on resistant bovines semi-engorged < 4 mm; (4) Male salivary gland from tick fed on resistant bovines 17–21d post attachment; (5) Unfed larvae from tick fed on susceptible bovines; (6) Nymph salivary gland from tick fed on susceptible bovines 11–14d post attachment; (7) Female salivary gland from tick fed on susceptible bovines semi-engorged < 4 mm; (8) Male salivary gland from tick fed on susceptible bovines 17–21d post attachment, respectively.

### Ethics statement

The study received approval of Animal Research Ethics Committee from Ribeirão Preto School of Medicine of University of São Paulo (protocol number 102/2009) for compliance with Ethical Principles in Animal Research adopted by Brazilian College of Animal Experimentation.

## Supplementary information

Supplementary file1 (XLSX 18760 kb)

Supplementary file2 (DOCX 26 kb)

Supplementary file3 (XLSX 47 kb)

## Data Availability

The sequences from *R. microplus* were deposited at GenBank (NCBI) as BioProject ID PRJNA329522 containing eight biosamples (SAMN05439577, SAMN05439580, SAMN05439579, SAMN05439578, SAMN05439573, SAMN05439576, SAMN05439575 and SAMN05439574).
