# Supplementary material for: A transcriptome and proteome of the tick Rhipicephalus microplus shaped by the genetic composition of its hosts and developmental stage
Source: Sci Rep. 2020 Jul 30;10:12857. doi: 10.1038/s41598-020-69793-3 (PMC7393499; doi:10.1038/s41598-020-69793-3)
Supplement: Supplementary file 2 — Supplementary file2 (DOCX 26 kb) [file 41598_2020_69793_MOESM2_ESM.docx]

**A transcriptome and proteome of the tick *Rhipicephalus microplus* shaped by the genetic composition of its hosts and developmental stage.**

Gustavo R. Garcia, José Marcos Ribeiro, Sandra Regina Maruyama, Luiz Gustavo Gardinassi, Kristina Nelson, Beatriz R. Ferreira, Thales Galdino Andrade, and Isabel K. Ferreira de Miranda Santos.

**Additional File 2. Functional classification of transcripts from the *R. microplus****.*

| ***Functional classification of* R. microplus *transcripts of the secreted category*** | | | | |  |
| --- | --- | --- | --- | --- | --- |
| **Class** | **N° of CDS** | **N° of Reads** | **% of total reads^*^** | **Reads/CDS** | **Match with proteomes?** |
| PROTEINASE INHIBITOR DOMAINS |  |  |  |  |  |
| KUNITZ DOMAINS |  |  |  |  |  |
| Hexalaris | 2 | 68 | 0.02 | 34 |  |
| Pentalaris | 4 | 136 | 0.05 | 34 |  |
| Tetralaris | 11 | 345 | 0.12 | 31.36 |  |
| Trilaris | 28 | 920 | 0.33 | 32.86 |  |
| Bilaris | 61 | 1,598 | 0.57 | 26.20 |  |
| Monolaris | 95 | 2,341 | 0.84 | 24.64 |  |
| TIL domain (may have anti-microbial activity) | 72 | 2,260 | 0.81 | 31.39 |  |
| Thyropin domains | 4 | 76 | 0.03 | 19.00 |  |
| Cystatin | 34 | 941 | 0.34 | 27.68 |  |
| Serpin | 15 | 954 | 0.34 | 63.60 | Y |
| Kazal domain containing peptides | 6 | 306 | 0.11 | 51.00 |  |
| Carboxypeptidase inhibitor | 17 | 508 | 0.18 | 29.88 |  |
| Chimadanin | 4 | 491 | 0.18 | 122.75 |  |
| Phosphatidylethanolamine-binding protein | 1 | 31 | 0.01 | 31.00 |  |
| Other protease inhibitor | 14 | 527 | 0.19 | 37.64 |  |
| ENZYMES |  |  |  |  |  |
| SECRETED PEPTIDASES |  |  |  |  |  |
| Metalloproteases of the reprolysin family | 115 | 5,686 | 2.04 | 49.44 |  |
| Neprilysin-type metalloprotease |  |  |  |  |  |
| M13 family/neprilysin | 44 | 958 | 0.34 | 21.77 |  |
| Other metalloproteases | 13 | 339 | 0.12 | 26.08 |  |
| Dipeptidyl-peptidase / M2 family | 7 | 447 | 0.16 | 63.86 |  |
| Serine proteases | 74 | 5,494 | 1.97 | 74.24 | Y |
| Serine carboxypeptidases | 25 | 544 | 0.20 | 21.76 |  |
| Transglutaminase | 4 | 455 | 0.16 | 113.75 |  |
| Legumain family | 5 | 149 | 0.05 | 29.80 |  |
| Endopeptidases | 7 | 216 | 0.08 | 30.86 | Y |
| Cathepsin (aspartyl and cysteine proteases) | 4 | 103 | 0.04 | 25.75 | Y |
| Other peptidases | 4 | 75 | 0.03 | 18.75 |  |
| SECRETED NUCLEASES |  |  |  |  |  |
| Deoxyribonuclease II | 10 | 404 | 0.15 | 40.40 |  |
| dsRNA-specific ribonuclease | 1 | 11 | 0.00 | 11.00 |  |
| Ribonuclease. T2 family | 2 | 22 | 0.01 | 11.00 |  |
| Other endonucleases | 3 | 33 | 0.01 | 11.00 |  |
| 5' nucleotidase | 14 | 572 | 0.21 | 40.86 |  |
| LIPASES AND ESTERASES | 6 | 195 | 0.07 | 32.50 |  |
| SPHINGOMYELIN PHOSPHODIESTERASE | 2 | 18 | 0.01 | 9.00 |  |
| SECRETED GLYCOSIDASES | 15 | 456 | 0.16 | 30.40 |  |
| GALACTOSIDASE/FUCOSIDASE | 15 | 1,012 | 0.36 | 67.47 | Y |
| MUCINS | 215 | 21,301 | 7.65 | 99.07 | Y |
| LIPOCALINS/ HISTAMINE BINDING PROTEIN/ P27 | 294 | 10,524 | 3.78 | 35.80 | Y |
| ANTIGEN 5 FAMILY | 18 | 1,879 | 0.68 | 104.39 | Y |
| PROKINETICIN DOMAIN-CONTAINING PEPTIDES | 3 | 20 | 0.01 | 6.67 |  |
| SECRETED IMMUNITY RELATED PROTEINS |  |  |  |  |  |
| ANTIMICROBIAL PEPTIDES |  |  |  |  |  |
| Defensin | 22 | 944 | 0.34 | 42.91 |  |
| Longicornsin | 4 | 126 | 0.05 | 31.50 |  |
| Hebreain/ricinusin/microplusin family | 9 | 679 | 0.24 | 75.44 |  |
| Neutrophil elastase proteins inhibitor | 5 | 528 | 0.19 | 105.60 | Y |
| Other intermediate proteins | 13 | 281 | 0.10 | 21.62 |  |
| Lysozyme | 3 | 32 | 0.01 | 10.67 |  |
| IMMUNOGLOBULIN BINDING PROTEINS | 12 | 2,810 | 1.01 | 234.17 | Y |
| DAP-36 IMMUNOSUPPRESSANT FAMILY | 16 | 468 | 0.17 | 29.25 |  |
| EVASINS | 36 | 1,114 | 0.40 | 30.94 |  |
| PATHOGEN RECOGNITION PROTEINS |  |  |  |  |  |
| Ixoderin/ficolin | 11 | 277 | 0.10 | 25.18 |  |
| ML - Niemann-Pick family | 13 | 305 | 0.11 | 23.46 |  |
| TICK SPECIFIC PROTEINS |  |  |  |  |  |
| GLYCINE RICH FAMILY |  |  |  |  |  |
| Cuticle like proteins | 14 | 357 | 0.13 | 25.50 |  |
| Cement proteins | 49 | 27,527 | 9.89 | 561.78 | Y |
| Elastin proteins | 5 | 243 | 0.09 | 48.60 |  |
| Glue proteins | 5 | 217 | 0.08 | 43.40 |  |
| Silk-like proteins | 2 | 45 | 0.02 | 22.50 |  |
| GYG repeat-containing protein | 10 | 145 | 0.05 | 14.50 |  |
| GGY protein | 11 | 311 | 0.11 | 28.27 |  |
| Other glycine rich proteins | 84 | 12,590 | 4.52 | 149.88 | Y |
| SALP-15 FAMILY | 3 | 88 | 0.03 | 29.33 |  |
| BASIC TAIL SUPERFAMILY | 23 | 801 | 0.29 | 34.83 |  |
| ACID TAIL SUPERFAMILY | 6 | 400 | 0.14 | 66.67 |  |
| 8.9 KDA FAMILY | 17 | 435 | 0.16 | 25.59 |  |
| 9.4 KDA FAMILY | 6 | 441 | 0.16 | 73.50 |  |
| 10 KDA FAMILY | 4 | 7,478 | 2.69 | 1869.50 |  |
| SIMILAR TO Rhipicephalus |  |  |  |  |  |
| Related to *R. microplus* proteins | 243 | 11,045 | 3.97 | 45.45 | Y |
| Related to *R. appendiculatus* proteins | 113 | 5,089 | 1.83 | 45.04 | Y |
| SIMILAR to Amblyomma |  |  |  |  |  |
| Related to *Amblyoma maculatum* proteins | 49 | 1,294 | 0.46 | 26.41 |  |
| Related to *Amblyomma variegatum* proteins | 6 | 101 | 0.04 | 16.83 |  |
| Related *to Amblyomma americanum* proteins | 6 | 86 | 0.03 | 14.33 |  |
| SIMILAR to *Ixodes scapularis* | 12 | 918 | 0.33 | 76.50 | Y |
| SIMILAR to *Dermacentor andersoni* | 45 | 1,148 | 0.41 | 25.51 |  |
| OTHER SECRETED PROTEINS | 43 | 1,409 | 0.51 | 32.77 | Y |
| OTHER UNCHARACTERIZED PUTATIVE SECRETED PROTEINS | 1,432 | 136,204 | 48.93 | 95.11 | Y |
| **Total of secreted category** | **3,600** | **278,351** |  |  |  |
| ***Functional classification of R. microplus transcripts of housekeeping category*** | | | | |  |
| **Class** | **N° of CDS** | **N° of Reads** | **% of total reads^#^** | **Reads/CDS** | **Match in proteomes?** |
| IMMUNITY RELATED PRODUTCS |  |  |  |  |  |
| Immunoglobulin G binding protein | 14 | 3,795 | 1.24 | 271.1 | Y |
| Similar to macrophage migration inhibitory factor | 7 | 263 | 0.09 | 37.6 |  |
| Similar to protein associated with Interferon | 7 | 243 | 0.08 | 34.7 |  |
| Toll-like receptors | 2 | 121 | 0.04 | 60.5 | Y |
| Alpha-2-macroglobulin | 4 | 89 | 0.03 | 22.3 |  |
| Other proteins possibly associated with immunity | 18 | 867 | 0.28 | 48.2 |  |
| PEPTIDASES |  |  |  |  |  |
| Cathepsin (aspartyl and cysteine proteases) | 7 | 368 | 0.12 | 52.6 |  |
| Calpain | 3 | 41 | 0.01 | 13.7 |  |
| Aminopeptidase | 6 | 125 | 0.04 | 20.8 |  |
| Membrane protease | 5 | 224 | 0.07 | 44.8 |  |
| signal peptide peptidase | 4 | 168 | 0.05 | 42.0 |  |
| Carboxypeptidase | 6 | 329 | 0.11 | 54.8 |  |
| Other peptidases | 11 | 246 | 0.08 | 22.4 | Y |
| PROTEIN EXPORT MACHINERY | 245 | 11,135 | 3.62 | 45.4 | Y |
| CYTOSKELETAL | 284 | 14,934 | 4.86 | 52.6 | Y |
| DETOXIFICATION |  |  |  |  |  |
| Sulfotransferases | 16 | 441 | 0.14 | 27.6 |  |
| Glutathione transferase | 16 | 292 | 0.10 | 18.3 | Y |
| Dehydrogenases | 19 | 274 | 0.09 | 14.4 |  |
| O-Methyl transferases | 3 | 18 | 0.01 | 6.0 |  |
| Multidrug resistance transporters | 4 | 71 | 0.02 | 17.8 |  |
| Oxidative detoxification |  |  |  |  |  |
| Cytochrome P450 | 24 | 519 | 0.17 | 21.6 | Y |
| Catalase | 7 | 115 | 0.04 | 16.4 |  |
| Superoxide dismutase | 4 | 45 | 0.01 | 11.3 |  |
| Selenoproteins | 6 | 188 | 0.06 | 31.3 |  |
| EXTRACELLULAR MATRIX AND ADHESION | 153 | 18,777 | 6.11 | 122.7 | Y |
| METABOLISM |  |  |  |  |  |
| Amino acid | 59 | 3,145 | 1.02 | 53.3 | Y |
| Carbohydrate | 76 | 3,835 | 1.25 | 50.5 | Y |
| Energy | 193 | 47,209 | 15.37 | 244.6 | Y |
| Intermediate | 39 | 833 | 0.27 | 21.4 | Y |
| Lipid | 144 | 3,694 | 1.20 | 25.7 | Y |
| Nucleotide | 78 | 2,772 | 0.90 | 35.5 | Y |
| NUCLEAR EXPORT | 11 | 478 | 0.16 | 43.5 | Y |
| NUCLEAR REGULATION | 136 | 4,633 | 1.51 | 34.1 | Y |
| PROTEIN MODIFICATION MACHINERY | 220 | 11,878 | 3.87 | 54.0 | Y |
| PROTEASOME MACHINERY | 139 | 5,263 | 1.71 | 37.9 | Y |
| PROTEIN SYNTHESIS MACHINERY | 312 | 30,173 | 9.82 | 96.7 | Y |
| SIGNAL TRANSDUCTION | 522 | 19,438 | 6.33 | 37.2 | Y |
| Similar to protein associated with Apoptosis | 42 | 2,070 | 0.67 | 49.3 |  |
| TRANSCRIPTION FACTORS | 93 | 5,258 | 1.71 | 56.5 | Y |
| TRANSCRIPTION MACHINERY | 456 | 26,075 | 8.49 | 57.2 | Y |
| TRANSPORTERS AND STORAGE | 214 | 8,966 | 2.92 | 41.9 | Y |
| UNKNOWN CONSERVED PROTEINS | 988 | 76,328 | 24.85 | 77.3 | Y |
| UNKNOWN CONSERVED SECRETED PROTEINS | 32 | 1,448 | 0.47 | 45.3 | Y |
| **Total of housekeeping category** | **4,629** | **307,184** |  |  |  |

The asterisk symbol (*) represents the total of reads from secreted category and the hash symbol (#) represents the total of reads from housekeeping category. The (Y) represents yes or the family presence in corresponding proteomes.
